# Heterogeneity of Microbial Communities on Deep-Sea Ferromanganese Crusts in the Takuyo-Daigo Seamount

**DOI:** 10.1264/jsme2.ME18090

**Published:** 2018-10-30

**Authors:** Shingo Kato, Tomoyo Okumura, Katsuyuki Uematsu, Miho Hirai, Koichi Iijima, Akira Usui, Katsuhiko Suzuki

**Affiliations:** 1 Ore Genesis Research Unit, Project Team for Development of New-generation Research Protocol for Submarine Resources, Japan Agency for Marine-Earth Science and Technology (JAMSTEC) Yokosuka, Kanagawa, 237–0061 Japan; 2 Research and Development Center for Submarine Resources, JAMSTEC Yokosuka, Kanagawa, 237–0061 Japan; 3 Department of Subsurface Geobiological Analysis and Research, JAMSTEC Yokosuka, Kanagawa, 237–0061 Japan; 4 Center for Advanced Marine Core Research, Kochi University Nankoku, Kochi, 738–8502 Japan; 5 Department of Marine & Earth Sciences, Marine Works Japan, Ltd. Yokosuka, Kanagawa 237–0061 Japan; 6 Research and Development Center for Marine Biosciences, JAMSTEC Yokosuka, Kanagawa, 237–0061 Japan

**Keywords:** deep-sea ferromanganese crusts, microbial community, biogeochemical cycling, 16S rRNA gene analysis

## Abstract

Rock outcrops of aged deep-sea seamounts are generally covered with Fe and Mn oxides, known as ferromanganese (Fe–Mn) crusts. Although the presence of microorganisms in Fe–Mn crusts has been reported, limited information is currently available on intra- and inter-variations in crust microbial communities. Therefore, we collected several Fe–Mn crusts in bathyal and abyssal zones (water depths of 1,150–5,520 m) in the Takuyo-Daigo Seamount in the northwestern Pacific, and examined microbial communities on the crusts using culture-independent molecular and microscopic analyses. Quantitative PCR showed that microbial cells were abundant (10^6^–10^8^ cells g^−1^) on Fe–Mn crust surfaces through the water depths. A comparative 16S rRNA gene analysis revealed community differences among Fe–Mn crusts through the water depths, which may have been caused by changes in dissolved oxygen concentrations. Moreover, community differences were observed among positions within each Fe–Mn crust, and potentially depended on the availability of sinking particulate organic matter. Microscopic and elemental analyses of thin Fe–Mn crust sections revealed the accumulation of microbial cells accompanied by the depletion of Mn in valleys of bumpy crust surfaces. Our results suggest that heterogeneous and abundant microbial communities play a role in the biogeochemical cycling of Mn, in addition to C and N, on crusts and contribute to the extremely slow growth of Fe–Mn crusts.

The seafloor covers nearly 70% of the Earth’s surface, with most of the seafloor being at a water depth of 1,000–6,000 m (bathyal and abyssal zones). Although the deep seafloor is mostly covered by sediments that limit seawater interactions with crustal materials, rock outcrops on ridges and at the slopes of seamounts are constantly and directly exposed to the overlying bottom seawater. Outcrops on the slopes of aged and volcanically-inactive seamounts at the bathyal and abyssal zones are commonly covered with iron (Fe) and manganese (Mn) oxides, known as ferromanganese crusts (Fe–Mn crusts) (17, 23, 68). Another form of Fe–Mn oxide on the seafloor is the spherical ferromanganese nodules on abyssal seafloor sediments (17, 23). These Fe–Mn oxides adsorb dissolved metallic ions from seawater (28, 32), and, thus, are referred to as the “chemical scavengers of the sea” (18). Due to the high concentrations of associated economically valuable elements (such as Ti, Co, Ni, and Te), Fe–Mn crusts and nodules are of economic interest as potential mineral resources (22, 50). Fe and Mn oxides in Fe–Mn crusts accumulate very slowly (1–10 mm per million years (Myr)) from overlying seawater on the surfaces of rock outcrops (17, 20, 23). Crusts that are thicker than 10 cm have been found in aged seamounts in the western Pacific (67–69). However, the growth mechanisms and biological contribution of Fe–Mn crusts remain unclear (32, 69).

The large-scale Cretaceous seamounts in the western Pacific provide unique opportunities for the study of microbial communities that have long been associated with outcrops (several tens of millions of years) along a wide range of water depths. The Takuyo-Daigo Seamount is a guyot (*i.e.*, flat-top seamount) located in the northwestern Pacific and has been extensively examined as a model seamount in various scientific fields, such as geology, geochemistry, and geomicrobiology (1, 19, 38, 42–44, 66, 69). This seamount is located on one of the oldest oceanic plates (>150 Myr old [39, 41]) and is in a region with low photosynthetic productivity (3). Thus, organic carbon availability on the seamount may be low and will affect the microbial community. The age of the guyot is estimated to be 100 Myr old (66). The flat top of the guyot is located at a water depth of 810 m, elevated by approximately 6,000 m from the abyssal seafloor. Fe–Mn crusts have been found almost continuously on the slope at a water depth of up to 5,500 m at the guyot (69).

To date, 16S rRNA gene analyses have been performed on Fe–Mn crusts collected in the Takuyo-Daigo Seamount at only bathyal zones (water depths of 1,200–3,000 m) (42, 43). Previous studies revealed that bathyal Fe–Mn crusts harbor diverse and abundant microorganisms including *Gammaproteobacteria*, *Alphaproteobacteria*, and *Thaumarchaeota*, and that the microbial community composition of Fe–Mn crusts is distinct from that of bottom seawater. However, information on microbial communities on Fe–Mn crusts is still limited by inadequate sampling and analyses; *e.g.*, no abyssal samples have been examined, only a subsample from each sample has been analyzed, and a small number (70–100) of PCR clones have been sequenced. Therefore, several issues remain unclear. The first question is which microorganisms are present as abundant and rare members in Fe–Mn crusts at abyssal zones. Accordingly, the second question is whether microbial communities on Fe–Mn crusts change with water depths from the bathyal to abyssal zones, as has been observed for the deep-sea water column (6, 35, 46, 58, 71) and deep-sea surface sediments (4, 11). The third question is how heterogeneous microbial communities are within a Fe–Mn crust. We hypothesize that the diversity and composition of microbial communities on Fe–Mn crusts are constrained by environmental factors (such as pressure, oxygen concentrations, and nutrient availability) through the water depths at a seamount. We also propose that the diversity and composition of microbial communities differ based on their localization within a crust. However, the above hypotheses have not been supported nor assessed by previous studies with limited datasets (42, 43). To address the above questions, we revisited the Takuyo-Daigo Seamount, collected Fe–Mn crusts at both the abyssal and bathyal zones, and performed culture-independent molecular biological analyses and microscopic observations. The present results will expand our understanding of the microbial ecology of seafloor outcrops.

## Materials and Methods

### Field sampling

Samples of Fe–Mn crusts were collected at water depths of 1,150–5,520 m (Fig. S1) in the Takuyo-Daigo Seamount (Fig. S2) during the KR16-01 cruise (January 2016) with the research vessel (R/V) Kairei (JAMSTEC, Yokosuka, Kanagawa, Japan) and the remotely operated vehicle (ROV) Kaiko Mk-IV (JAMSTEC). A conductivity-temperature-depth (CTD)—dissolved oxygen (DO) sensor (SBE-49 FastCAT, Sea-Bird Electronics, Bellevue, WA, USA) equipped with the ROV was used to measure the conductivity, temperature, salinity, and DO concentration of water columns overlying the seamount. The samples used in the present study are listed in Table S1. In the microbiological analysis, the samples collected were placed into a sealable box using the manipulators of the ROV on the seafloor to avoid contamination from surface seawater or sediments stirred up during other operations. The box was filled with ultra-pure water before the dives and opened only during sample recovery at each sampling site.

**Fig. 1:**
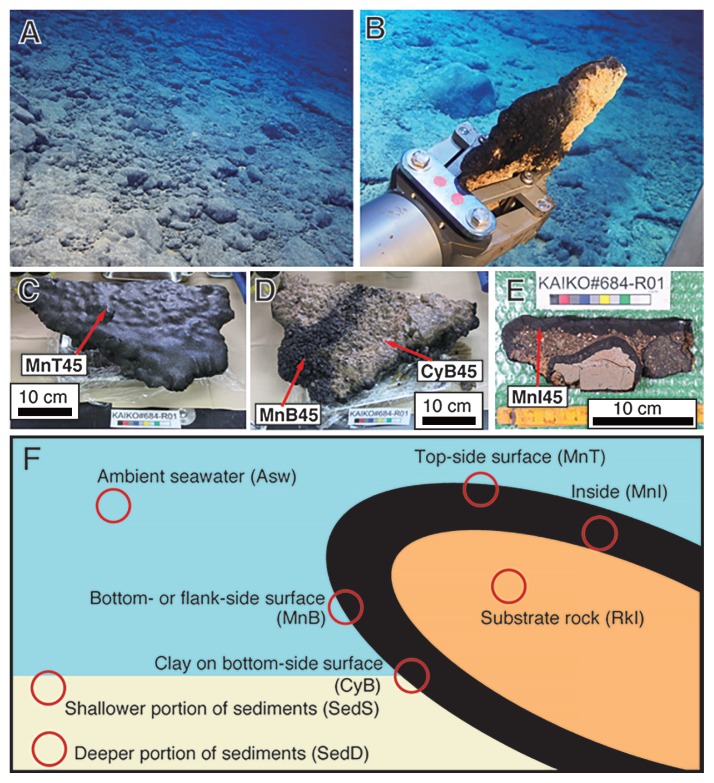
Representative photos of Fe–Mn crusts and sampling positions. (A) An on-site photo during Dive#684 at a water depth of 4,480 m. Round-and slab-shaped crusts with grayish white sandy thin sediments were observed. (B) Sampling a slab-shaped crust sample (684R1) using a manipulator during Dive#684. (C, D, and E) Photos of the top side, bottom side, and cross-section of the crust (684R1). Notably, two rocks with Fe–Mn crusts were observed inside the sample in panel (E), indicating that Fe–Mn oxides previously grew on the two rocks, rubble covered the rocks once, and the growth of Fe–Mn oxides then re-started on the covered rubble. (F) Conceptual illustration of sampling positions in each sample, *i.e.*, Fe–Mn crust, sediment, and ambient bottom seawater. Photos for other dives and other crust samples are shown in Fig. S1 and S4.

On board, collected samples were treated as described previously (42, 43) with minor modifications. To assess the variability in microbial communities within a crust sample, we subsampled ~1-cm^3^ fragments of the top surface (subsample ID with “MnT”) with grayish white sands, bottom surface (“MnB”) without particles, and interior (~2 cm from the surface, “MnI”) of Fe–Mn crust samples (Fig. 1). Clayish materials (“CyB”) attached to the bottom sediments were also subsampled from some samples (Fig. 1). The subsamples of MnT, MnB, and MnI were gently washed with filter-sterilized seawater three times to remove loosely attached particles that are potential contaminants from bottom seawater and sediments. In two out of the 4 sediment samples, shallower (0–5 cm from the surface; SedS) and deeper (5–10 cm from the surface; SedD) parts were subsampled using sterile spatulas (Fig. 1). Some subsamples were stored at −80°C for the DNA analysis. Regarding microscopy, some subsamples were fixed in filtered seawater with glutaraldehyde (final concentration, 2%) or formaldehyde (final concentration, 3.7%) at 4°C overnight, gently washed three times with filtered seawater, and stored in PBS/EtOH (1:1) solution at −20°C.

### 16S rRNA gene amplicon sequencing

DNA was extracted from the subsamples (0.3–0.8 g) using a FastDNA SPIN kit for soil (MP Biomedicals, Santa Ana, CA, USA) with a FastPrep instrument (MP Biomedicals). Partial 16S rRNA genes (V4 region) were amplified by PCR with the prokaryote-universal primer set, 515F (29) and 806R (9). Amplicon sequencing was performed using an Illumina HiSeq 2500 platform (250-bp paired-end). PCR and sequencing were performed by Novogene (Beijing, China).

### Sequence analysis

The merging, quality filtering, chimera removal, and operational taxonomic unit (OTU) clustering with 97% similarity of raw paired-end reads were performed using the UPARSE pipeline (14). The taxonomic affiliations of the OTUs were assessed using QIIME version 1.9.1 (8) against SILVA database release 128 (47). OTUs affiliated with mitochondria, chloroplasts, *Eukarya*, and unidentified (possibly non-SSU rRNA genes) were removed from subsequent analyses.

Analyses of alpha diversity (*i.e.*, Chao1 species richness estimates, Shannon and Simpson diversity indices, and rarefaction curves) and beta diversity (*i.e.*, non-metric multidimensional scaling [NMDS] and canonical correspondence analysis [CCA]) based on the Bray-Curtis dissimilarity index were performed in R version 3.3.3 (https://www.R-project.org) using Phyloseq version 1.19.1 (37). In the CCA, the significance of constraints was assessed by an analysis of variance (ANOVA) with 999 permutations in R package vegan version 2.4-4 (https://cran.r-project.org/package=vegan). NMDS and CCA were performed for abundant (≥1% of the total reads of each sample), rare (<1%), and all OTUs, respectively. Rare OTUs were not expected to be detected based on a previous PCR clone analysis with approximately 100 clones analyzed (42, 43). Non-parametric statistics, *i.e.*, Spearman’s rank correlation coefficient and the Wilcoxon rank-sum test, were conducted in R.

Nucleotide sequences were aligned using MUSCLE version 3.8.31 with the default parameters (13). The alignment was trimmed using trimAl version 1.2rev59 with the option ‘-automated1’ (7). Maximum likelihood trees were constructed using RAxML version 8.2.9 (60) with the GTRGAMMA model.

### Q-PCR

The copy numbers of prokaryotic or archaeal 16S rRNA genes were evaluated by quantitative PCR (Q-PCR) as previously described (46). Aliquots of the same DNA extracts used in the above amplicon sequencing were employed. Briefly, the prokaryote-universal primer-probe set, Uni340F–Uni516F (TaqMan probe)–Uni806R, or archaea-specific primer-probe set, Arch349F–Arch516F (TaqMan probe)–Arch806R, was used. The copy numbers of each gene were quantified as the average of at least triplicate analyses.

### Cryo-thin sectioning and fluorescent microscopy

The cryo-thin sectioning and fluorescent microscopy of subsamples of Fe–Mn crust surface samples were performed as previously described (57) with some modifications. Fixed subsamples with formaldehyde were soaked in mounting medium (SCEM, Muto Pure Chemicals, Tokyo, Japan) at 4°C overnight, frozen at −25°C, and then cut into ~5-μm-thick sections using a cryostat (CM1520, Leica, Wetzlar, Germany). We carefully selected the cutting face to obtain vertical cross-sections including the surface, and used an adhesive film (Cryofilm Type IIC, Leica) to minimize the mechanical damage caused by sectioning (30). After gently rinsing mounting medium with sterilized PBS, the microbial cells in cryo-sections were stained with SYBR Green I and observed by fluorescence microscopy (BX60, Olympus, Tokyo, Japan).

### Scanning electron microscopy and energy-dispersive X-ray spectroscopy

Samples fixed with 2.5% glutaraldehyde were washed in filtered seawater, and post-fixed with 2% osmium tetraoxide in filtered seawater at 4°C for 2 h. Samples were rinsed with distilled water, and conductive staining was then performed by incubating 1% aqueous tannic acid (pH 6.8) for 60 min. Stained samples were washed with distilled water and treated with 1% aqueous osmium tetraoxide for 60 min. Samples were dehydrated with a graded ethanol series (70, 80, 90, and 99% ethanol) and subjected to critical point drying (JCPD-5; JEOL, Tokyo, Japan). Samples were mounted on stubs and coated with osmium using an osmium plasma coater (POC-3; Meiwafosis, Osaka, Japan). Coated samples were observed by field-emission scanning electron microscopy (SEM) (JSM-6700F, JEOL) at an acceleration voltage of 5 keV. An elemental analysis using energy dispersive X-ray spectrometry (EDS) (JED 2300, JEOL) equipped with SEM was performed at an acceleration voltage of 15 keV. Fixed and cryo-sectioned samples were dehydrated with a graded ethanol series, air-dried, and observed by SEM-EDS as described above.

### Accession numbers

The raw sequence data obtained in the present study have been deposited into the DNA Data Bank of Japan (DDBJ) under the accession numbers DRA006508 for the 16S RNA gene amplicon sequences. The taxonomic affiliations and nucleotide sequences of the OTUs have been deposited into FigShare (https://figshare.com/articles/Appendix_data_xlsx/6484025).

## Results

### Site description and sampling

During our cruises with the ROV, Fe–Mn crusts were observed at the shoulder of the flat top at a water depth of 1,100 m through to nearly the foot of the seamount at 5,500 m (Fig. 1 and S1) of the southern ridge of the Takuyo-Daigo Seamount (Fig. S2) as reported previously (69). In many cases, grayish or whitish sand slightly covered the flat or gentle slope of the surfaces of the crusts at each depth (Fig. 1A and S1). The crusts were slab- or round-shaped, which originated from the shape of the substrates, such as volcanic and calcareous rocks (Fig. S1). The water depth profile for temperature, salinity, and DO concentration is shown in Fig. S3, which is consistent with previous findings for shallower than 3,000 m (43). An oxygen minimum zone was observed at water depths of 900–1,000 m with 20% of the saturated DO concentration. Salinity and temperature were constant from water depths of 3,000 to 5,500 m (34.5 practical salinity unit and 2–3°C), whereas DO concentrations slightly increased from 44 to 51% of the saturated concentration.

As reported previously (69), the top- and bottom-side surfaces of some crust samples had a bumpy appearance (Fig. 1C, D, and S4). Sandy materials were observed on the top-side surface of some crust samples (678R1, 679R1, and 682R2; Fig. S4) and were not detached by repeated rinsing and inverting in filtered seawater. Thus, sandy materials adhered to the surface. In contrast, sandy materials were not observed on the bottom-or flank-side surface of the crust, which was not attached to the seafloor sediments. Clay-like materials were observed on the bottom-side surface of some crust samples (679R1, 682R3, 683R7, and 684R1; Fig. 1D and S4).

### Cell morphology and localization associated with elemental distribution

**Fig. 2:**
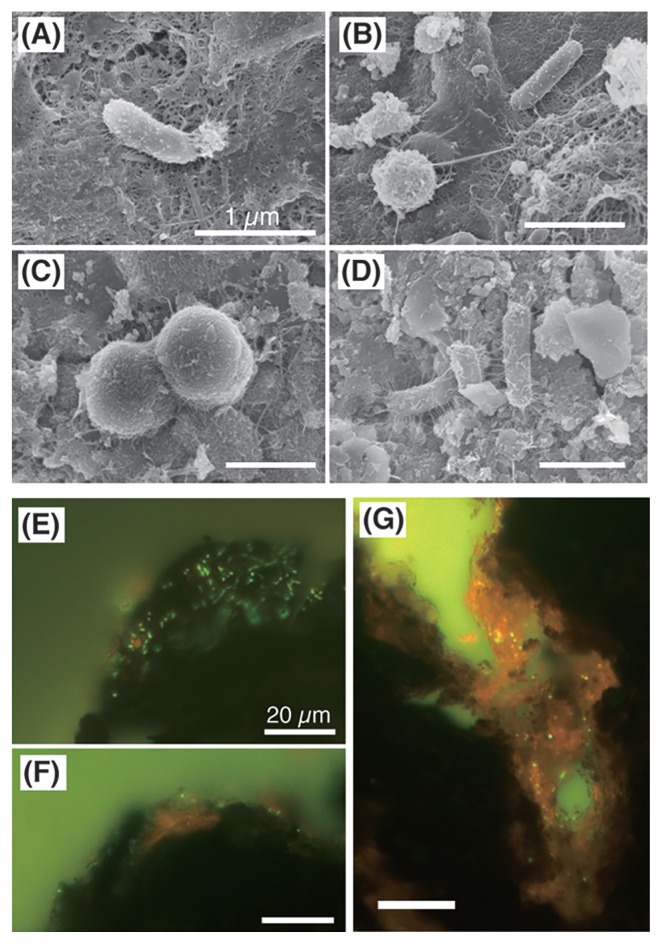
Microscopic images of microbes on crusts. Representative images of cocci- and rod-shaped microbial cells on surfaces of Fe-Mn crust samples (A) 678R1, (B) 679R1, (C) 682R3, and (D) 684R1 by scanning electron microscopy. Scale bars, 1 μm. (E, F, and G) Representative images of fluorescent microscopy of a thin section of the 684R1 sample. Cells were stained with SYBR Green I. Scale bars, 20 μm. (E and F) Cells (green or yellowish dots) on blackish Fe-Mn oxides of the crust surface. (G) Cells in orangish Fe oxides in the valley of the surface of crusts.

SEM revealed cocci- and rod-shaped microbial cells on the surfaces of Fe–Mn crust samples at each water depth (Fig. 2A, B, C, D, S5A, B, D, G, H, J, K, and L). These cells adhered to the surface via tens-nm-wide fibers. The nano-fibers reticulately covering the crust surface were often observed in several samples. Furthermore, large filamentous organisms (>10 μm in width, >1 mm in length in some cases) were observed on the surfaces of some crust samples by SEM (Fig. S5A, D, E, and K; see supplemental text for more details).

**Fig. 3:**
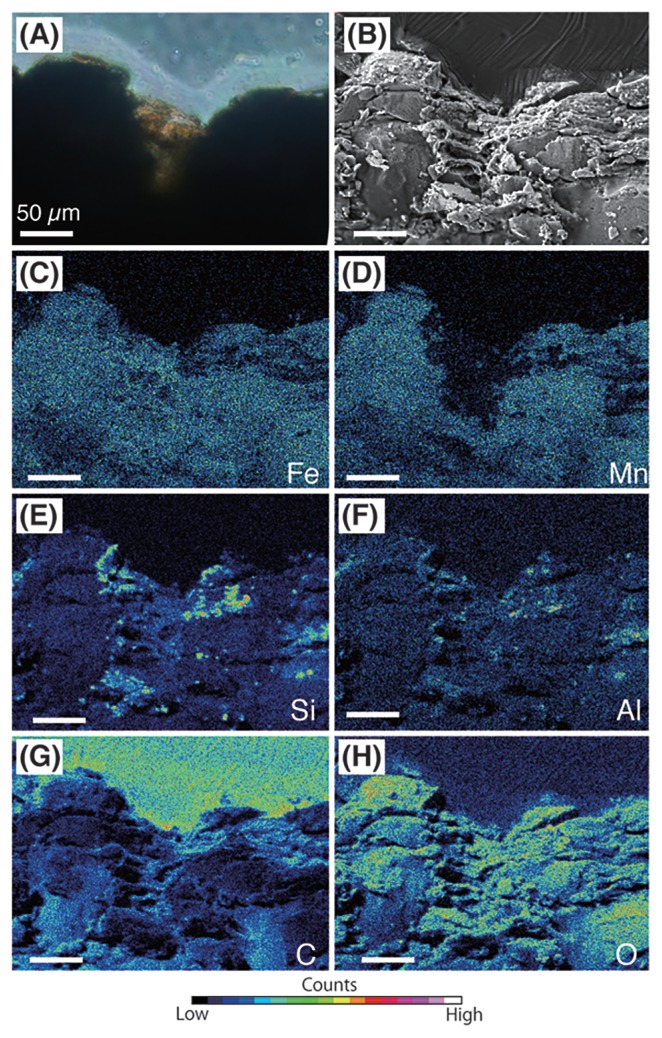
Representative result of correlative light and electron microscopy of a thin section of the crust sample (684R1). (A, B, C, D, E, F, G, and H) Upper and bottom sides of the images were seawater-side and crust-inside, respectively. Images of the same view point. Scale bars, 50 μm. (A) Light microscopy and (B) SEM. EDS mapping images of (C) iron, (D) manganese, (E) silicon, (F) aluminum, (G) carbon, and (H) oxygen.

Fluorescence microscopy of thin sections showed the spatial distribution of microbial cells on the crust surface (Fig. 2E, F, and G). The dense accumulation of cells was observed within several tens of micrometers on the surface. In contrast, cells were rarely observed in the interior of the crusts, *i.e.*, within Fe–Mn oxides, which is similar to a previously reported ferromanganese nodule (57); however, cell-sized (~1 μm in diameter) hollows were observed in the interior of the crusts by SEM (Fig. S5F and I). Microbial cells were concentrated with orangish or brownish materials in the valleys of bumpy surfaces (Fig. 2G) in some cases. A SEM-EDS analysis of thin sections indicated that the materials in the valleys were lower in Mn, but not in Fe, than the surrounding Fe–Mn oxides (Fig. 3 and S6), which is consistent with previous findings (72). Particles with higher concentrations of Si and Al were patchily detected in the valleys and interiors of the crusts, indicating the incorporation of the particulate debris of diatoms, almino silicate sands, and/or muds during crust growth, as previously reported for ferromanganese nodules (57). These Si- and Al-rich debris were also observed on the curst surface (Fig. S7B and D).

### Microbial abundance

**Fig. 4:**
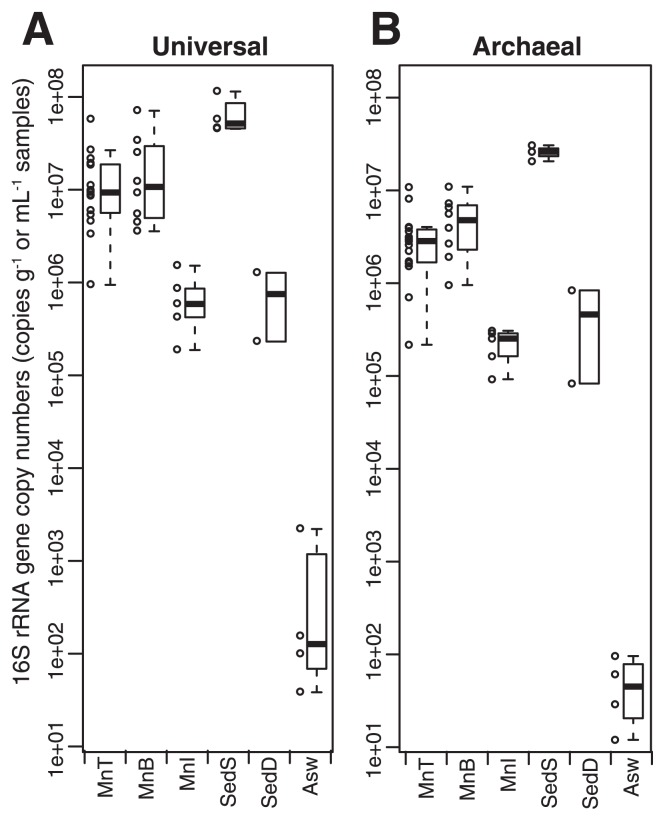
Q-PCR results. Box plot of (A) prokaryotic and (B) archaeal 16S rRNA gene copy numbers among habitat types. Circles beside the boxes represent actual values for all (sub)samples. A summary of the results obtained for all samples, except clay samples (*i.e.*, CyB), was shown.

The surfaces of the Fe–Mn crusts, even those at the abyssal zone, harbored abundant amounts of microbial cells. Q-PCR using prokaryote-universal and archaea-specific 16S rRNA gene primer sets (Table S1; Fig. 4) showed the abundance of archaeal and prokaryotic 16S rRNA genes in the samples. The top and bottom sides of the crust surface contained 0.1–7.2×10^7^ copies of prokaryotic 16S rRNA genes g^−1^ sample. 16S rRNA gene copy abundance varied between subsamples of each crust sample (*e.g.*, MnT45 and MnB45) from the same water depth, and even between biological duplicates at the same position (*e.g.*, MnT14a and MnT14b). The ratio of the number of archaeal 16S rRNA genes to that of prokaryotic 16S rRNA genes in crust surface samples (MnT and MnB) ranged between 9.2 and 71.2% (26.3% median; Table S1 and Fig. S8). There was no correlation between microbial abundance on the crust surface (top and bottom sides) and water depth (*n*=23; Spearman’s rho, >−0.12 and <0.34; *P*>0.2; Fig. S8).

Since the 16S rRNA gene copy number per cell was 1 for archaea and 3 for bacteria (61), total cell numbers were estimated to be in the order of 10^6^–10^8^ cells g^−1^. Gene abundance in the interior of the crusts (*i.e.*, MnI; *n*=5) was significantly lower (the Wilcoxon rank-sum test, *P*<0.05) than that in crust surface samples (*i.e.*, MnT and MnB; *n*=15, 8) (Fig. 4), indicating that fewer microbial cells were in the interior, which is consistent with microscopic results.

The microbial abundance of the crust surface (*i.e*., MnT and MnB; *n*=15, 8) was significantly greater than that of deeper sediments (*i.e.*, SedD; *n*=2) and seawater (Asw; *n*=4) (Wilcoxon rank-sum test, *P*<0.05), but less than that of shallower surface sediments (*i.e.*, SedS; *n*=4) (*P*<0.05) (Fig. 4). It is important to note that cell numbers estimated in ambient seawater (~10^2^ cells mL^−1^) from the copy number were one or two orders of magnitude lower than those in other deep-sea waters reported previously (27, 40, 46), and this may have been due to the low DNA extraction efficiency from membrane filters used to collect cells from seawater (Table S1). Thus, microbial abundance in ambient seawater samples may be underestimated. Nevertheless, the previously reported microbial abundance in seawater (10^3^–10^4^ cells mL^−1^) was still lower than that in crusts and sediments since the densities of Fe-Mn crusts and deep-sea sediments were generally <2 g mL^−1^ (25, 55).

### Microbial community composition and diversity

The deep-sequencing of 16S rRNA genes enabled us to analyze microbial communities including rare members (<1% of the total reads of each sample) that had been likely overlooked in previous PCR clone analyses using approximately 100 clones for each sample (43). Sequence data were successfully obtained from 22 crust (sub)samples, 2 clay samples, 6 sediment (sub)samples, and 6 seawater samples (total of 36 samples; Table S1). This produced a total of 1,985,013 merged paired-end reads. The filtering of low-quality reads, OTU clustering at 97% sequence similarity, and removal of potential chimeras and untargeted OTUs, such as eukaryotic, mitochondrial, and plastidic sequences, resulted in a total of 12,365 OTUs. Most OTUs (82.1% of the total) were classified into taxonomic groups without cultivated representatives at the genus or higher taxonomic levels.

Previous studies suggested that several sequences in genera, such as *Ralstonia* in *Betaproteobacteria*, *Pseudomonas* in *Gammaproteobacteria*, and *Bacillus* in *Firmicutes*, originated from contaminants in clean laboratory water and/or the reagents used in experimental procedures from DNA extraction to sequencing (33, 53). However, microorganisms in these genera have been isolated from oligotrophic marine environments, including basalts, sediments, and deep-sea water (48, 56, 65). Our samples were also from oligotrophic marine environments, and we found OTUs affiliated with these genera in our dataset. We used a negative control for DNA extraction and PCR amplification, and the amplification of gene fragments was not observed, even in second PCR. Thus, these OTUs may have originated from indigenous microorganisms in samples, and we did not exclude these OTUs from our dataset in further analyses.

### Alpha and beta diversities

The alpha diversity of crust surface communities appeared to decrease as water depth increased. We calculated Chao1 species richness estimates and Shannon and Simpson diversity indices using data normalized to the minimum number of reads (22,267 reads) (Fig. S9A) in order to compare alpha diversities among the habitats (MnT, MnB, CyB, SedS, SedD, and Asw; *n*=14, 8, 2, 4, 2, and 6, respectively). Four (sub)samples (MnT55b, MnT54a, Asw30, and Asw45) showed abnormally low values and seemingly biased microbial community structures, as described below. The results of the Wilcoxon rank-sum test for each pair of habitats without the above four samples are shown in Fig. S9B. Alpha diversity and rarefaction curves with non-normalized data are shown in Table S2 and Fig. S9C, respectively. Overall, the comparison indicated that (i) the alpha diversity of Fe–Mn crust samples was significantly lower than those of the other sample types and (ii) the alpha diversity of the top side of the crusts did not significantly differ from that of the bottom or flank side. When we included all crust surface samples (*i.e.*, MnT and MnB; *n*=22) in the analysis of the relationship between alpha diversity and water depths, Shannon and Simpson diversity indices correlated with water depth (Spearman’s rho=−0.51 and −0.65; *P*<0.02). Even when we excluded two of the crust surface samples from the abyssal zones (MnT55b and MnT54a) with abnormally low values, the Simpson diversity index still correlated with water depth (Spearman’s rho=−0.56; *P*<0.01).

**Fig. 5:**
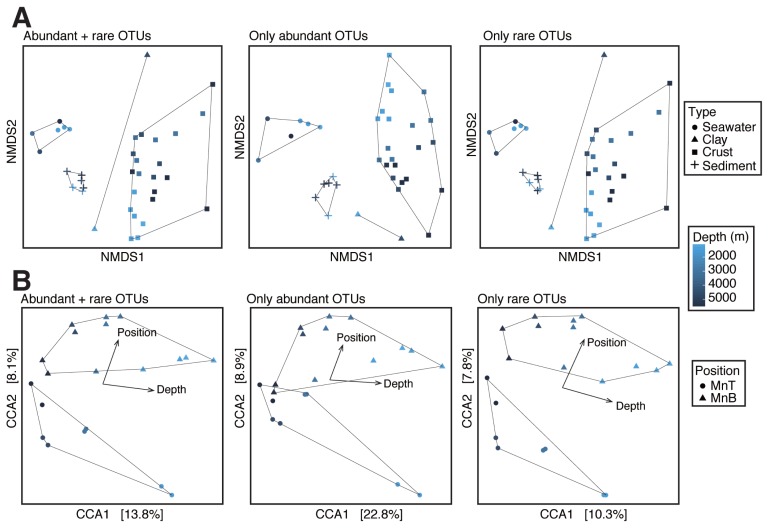
Beta diversity among habitat types and positions. (A) NMDS plot and (B) CCA based on the Bray-Curtis dissimilarity index. From left to right panels, results using all OTUs, only abundant OTUs (≥1% of the total reads of each sample), and only rare OTUs (<1%) are shown, respectively. Habitat types and positions are represented by the shapes of the markers indicated in the top- and bottom-right boxes. Water depths for the (sub) samples are represented by color gradation (from blue to black, and from shallow to deep) indicated in the middle-right box.

To visualize beta diversity among the samples, we performed NMDS based on the Bray-Curtis dissimilarity index. In an NMDS plot for whole communities (Fig. 5A), the samples of each habitat were clustered and then separated from each other roughly along the first NMDS axis. NMDS plots for abundant and rare sub-communities showed that abundant and rare sub-communities were both distinguishable among the habitats, as were whole communities. Furthermore, to evaluate the influence of water depths and sampling positions on Fe–Mn crust communities, we performed CCA for whole communities and abundant and rare sub-communities with the factors of depth and position (Fig. 5B). In this CCA, we used only crust samples (*i.e.*, MnT and MnB; *n*=20), except for two samples (MnT55b and MnT54a) that were anomalies in the NMDS plots and alpha diversity measurements. Samples were distinguishable depending on water depth (ANOVA, *P*<0.001) and position (*P*<0.05).

### Community composition at the population level

To overview the community structures of the samples, the relative abundance of taxonomic groups was summarized at the phylum or class level only for the phyla *Proteobacteria* (including the classes *Alpha*-, *Beta*-, *Delta*-, and *Gammaproteobacteria*) and *Thaumarchaeota* (including Marine Group I [MGI]) (Fig. S10A and B). In all samples, *Gammaproteobacteria*, *Alphaproteobacteria*, and MGI were relatively abundant (10–30% in most samples). These three taxa have also been detected as major taxa in oligotrophic deep-sea surface sediments and outcrops (34, 35, 43, 54, 58). The relative abundance of taxonomic groups at lower taxonomic levels in the three taxa is shown in Fig. S10C, D, E, F, G, and H. In MGI, the OTUs detected were classified into 10 clades (Fig. S10C and D) based on the phylogenetic tree (Fig. S11) and previous findings (12, 36). The relative abundance of less abundant taxa (<2.5% and <0.1% of the total OTUs for each sample) is shown in Fig. S12.

**Fig. 6:**
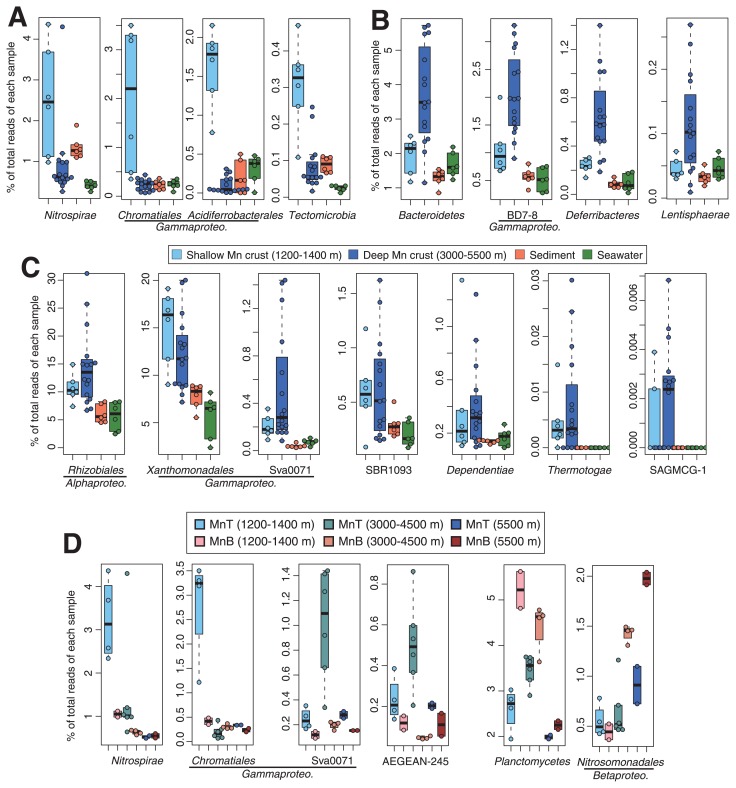
Box plot of relative abundance for representative taxa showing higher abundance in Fe–Mn crusts than in surrounding sediments and seawater. (A) Taxa highly abundant in shallower crusts than in deeper crusts. (B) Taxa highly abundant in deeper crusts than in shallower crusts. (C) Taxa showing similar abundance between shallower and deeper crusts. (D) Representative taxa showing significantly different abundance between the top- and bottom-side surfaces of the crusts. (A, B, C, and D) These data are representative of those shown in Fig. S10 and S12.

The relative abundance of several taxa was significantly greater (Wilcoxon rank-sum test, *P*<0.05) in shallow (1,200–1,400 m; *n*=6) and/or deep Fe–Mn crust samples (3,000–5,500 m; *n*=16) than in sediment (*n*=6) and seawater samples (*n*=6) (Fig. 6A, B, and C, which originated from Fig. S10 and S12). The relative abundance of *Nitrospirae*, *Chromatiales*, *Acidiferrobacterales*, and *Tectomicrobia* was significantly greater (*P*<0.001) in shallow Fe–Mn crust samples than in deep Fe–Mn crusts (Fig. 6A). In contrast, the relative abundance of *Bacteroidetes*, BD7-8, *Deferribacteres*, and *Lentisphaerae* was significantly greater (*P*<0.01) in deep Fe–Mn crust samples than in shallow Fe–Mn crusts (Fig. 6B).

In a comparison of the top and bottom sides of Fe–Mn crusts for water depths (MnT and MnB from water depths of 1,200–1,400, 3,000–4,500, and 5,500 m; *n*=2 to 6), the relative abundance of several taxa significantly differed (*P*<0.05) (Fig. 6D, which originated from Fig. S13). In shallower and deeper crust samples, the relative abundance of Sva0071 and AEGEAN-245 was greater in the top side than in the bottom side; in contrast, that of *Planctomycetes* and *Nitrosomonadales* was greater in the bottom side than in the top side. The relative abundance of *Nitrospirae* and *Chromatiales* was greater in the top side than in the bottom side for shallower samples only.

The relative abundance of the phyla *Woesearchaeota*, *Hydrogenedentes*, and *Zixibacteria* (Fig. S12B) and Eta, SAMGA-X, and Upsilon of the MGI clade (Fig. S10D) was significantly greater (*P*<0.005) in sediment samples than in other samples. The relative abundance of *Euryarchaeota* and *Marinimicrobia* (Fig. S10B), the Gamma-II of the MGI clades (Fig. S10D), SAR11 in *Alphaproteobacteria* (Fig. S10F), and *Oceanospirillales* and *Salinisphaerales* in *Gammaproteobacteria* (Fig. S10H) was significantly greater (*P*<0.01) in seawater samples than in other samples. The results obtained for relatively abundant taxa (>1% of the total reads in each sample), such as some MGI clades, SAR11, and *Oceanospirillales*, were consistent with those of a previous PCR clone analysis in this study area (43).

Some taxa were abnormally abundant in some samples, such as *Rhizobiales* in the Fe–Mn crust sample MnT54a (>45% of total reads), *Sphingomonadales* in the crust sample MnT55b (>40%), and *Alteromonadales* in the seawater samples Asw30 and Asw45 (>30%) (Fig. S10G). The colonies of these taxonomic members may have been non-homogeneous on the crust surface or in seawater as particles and we may have used samples containing these colonies by chance. The members of these taxa have been identified as major components of particle-attached microbial communities in deep seawater (16, 51). Therefore, we excluded these four samples from the dataset in some comparative analyses.

### Community composition at individual OTU levels

To assess core and abundant members only for Fe–Mn crust samples at the OTU level, we focused on OTUs detected in ≥70% of crust samples (≥14/20; two samples, MnT54a and MnT55b, with abnormally abundant taxa were excluded), which showed ≥10-fold higher medians of their relative abundance in crust samples than in other samples. Approximately 60 OTUs were relatively abundant in the Fe–Mn crust samples, but not in the other samples (Fig. S14). Five of these OTUs, *i.e.*, Otu92 in *Rhizobiales* of *Alphaproteobacteria*, Otu1017 in *Thermotogae*, Otu3936 in *Nitrosomonadales* of *Betaproteobacteria*, Otu873 in *Sphingobacteriia* of *Bacteroidetes*, and Otu1269 in *Nitrospirae*, were only detected in Fe–Mn crust samples (and clay samples).

Furthermore, more than 2,500 OTUs (26.9% of all OTUs detected in crust samples) were only detected in Fe–Mn crusts (Fig. S15A), indicating that Fe–Mn crusts harbored a large number of unique microorganisms. However, >97% of crust-unique OTUs were detected in <30% of crust samples (Fig. S15B), suggesting that the most unique members were not core members for the crusts through the water depths. These crust-unique OTUs were mainly affiliated with *Deltaproteobacteria* (particularly *Myxococcales*), *Gammaproteobacteria* (particularly unclassified clades at the order level), *Bacteroidetes*, and *Planctomycetes* (Fig. S15C and D). In unique OTUs for the other samples, *Firmicutes*, *Woesearchaeota*, and *Oligoflexales* were relatively abundant taxa in clay, sediment, and seawater samples, respectively. The presence of these unique OTUs in each habitat may have been one of the factors resulting in the differences observed in beta diversity among sample types (Fig. 5A).

**Fig. 7:**
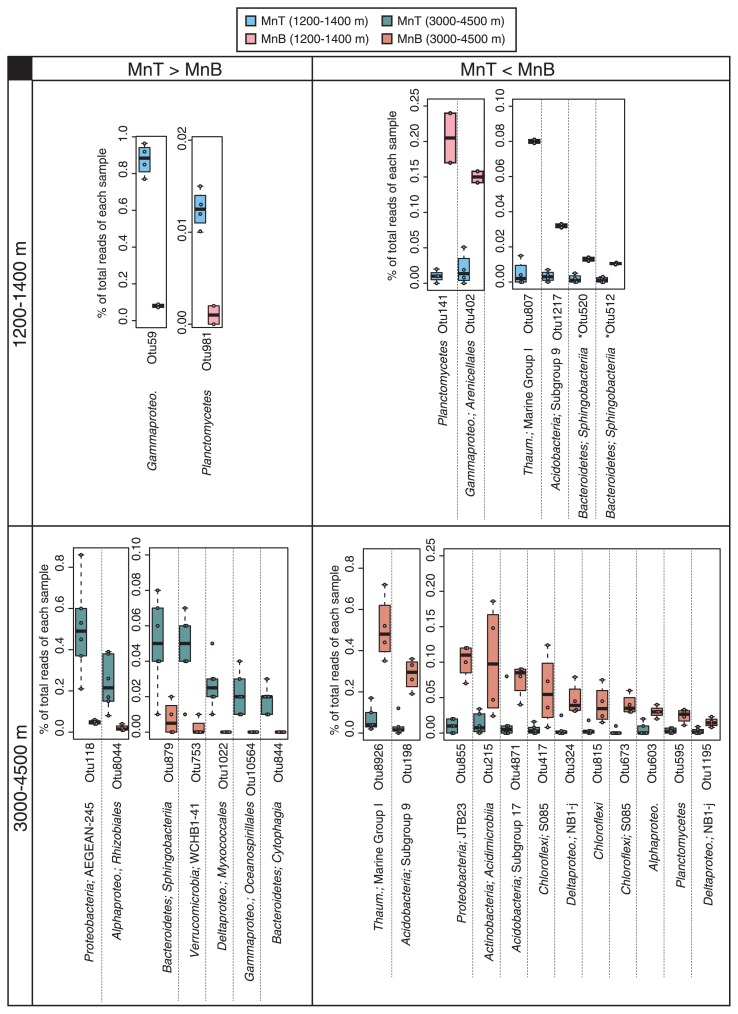
Box plot of relative abundance of individual OTUs showing higher abundance in shallower or deeper crusts collected at different water depths. The positions within a sample and water depths at which the samples were collected are represented by colors indicated in the top box. The taxonomic affiliation of each OTU is shown at the bottom of their ID. OTUs (Otu512 and Otu520) with asterisks as core OTUs for Fe–Mn crusts (Fig. S14; see text for details).

Comparisons between the top and bottom sides of the Fe–Mn crusts (*n*=4 and 2, respectively) revealed that the relative abundance of several OTUs significantly differed (>10-fold median, *P*<0.05) (Fig. 7). Samples from water depths of 5,400–5,500 m were excluded from comparisons because of the small number of samples (two for each side). Otu59 in *Gammaproteobacteria* and Otu981 in *Planctomycetes* were significantly more abundant in the top side of shallow crust samples than in the bottom side; in contrast, Otu141 in *Planctomycetes* and Otu402 in *Gammaproteobacteria* were more abundant in the bottom side than in the top side. Thus, even in the same taxonomic groups, a difference in relative abundance was observed at individual OTU levels. In deeper samples, some OTUs, such as Otu118 in AEGEAN-245 and Otu8044 in *Rhizobiales*, were greater in the top side than in the bottom side. In contrast, some OTUs, such as Otu8926 in MGI and Otu198 in *Acidobacteria*, were greater in the bottom side. In addition, there were numerous unique OTUs among the top and bottom sides of shallow and deep crust samples (Fig. S16). These OTUs may have contributed to the difference observed in beta diversity among crust samples (Fig. 5B). Of the OTUs showing significantly different abundance between the top and bottom side, only two OTUs (Otu512 and Otu520) in *Sphingobacteriia* and *Bacteroidetes* were core and abundant members in the crusts (Fig. S14).

## Discussion

### Potential for Mn release by microbial activity

The results of SEM-EDS analyses of cryo-thin sections imply that only Mn is released from Fe–Mn oxides in the valleys of the bumpy crust surface. The dense accumulation of cells was observed in the valleys by fluorescent microscopy. This result prompted us to speculate that the activity of accumulated microorganisms causes the release of Mn from the valleys. The Fe concentrations of orangish materials and the basement Fe-Mn crust were indistinguishable in SEM-EDS analyses (Fig. 3 and S6), which strongly supports the above speculation. A previous study suggested that microorganisms contribute to the dissolution of Mn in Fe–Mn nodules based on the detection of 16S rRNA genes related to known Mn-reducing bacteria (*i.e.*, *Shewanella* and *Colwellia*) as the majority (5). However, these 16S rRNA genes related to known Mn-reducing bacteria were rarely detected in the present study. A possible mechanism for microbial Mn release on the crust surface is acidification through the production of nitrite by ammonia oxidizers, organic acid production by fermenting microorganisms, or both. We detected 16S rRNA genes related to ammonia oxidizers, such as members of *Nitrosomonadales* and *Thaumarchaeota*, and fermenting microorganisms, including members of *Bacteroidetes*. The release of Mn by microorganisms may contribute to the extremely slow growth of Fe–Mn crusts. Even if Fe was also released, released Fe^2+^ may be rapidly oxidized and precipitated immediately under oxic conditions at the circumneutral pH of ambient seawater (49). However, we cannot exclude another possibility that Fe-rich and Mn-depleted pelagic clays stuffed the valleys. Even in this case, our results suggest that microorganisms contribute to sticking the clays to the valleys, which may affect the surface shape, growth rate, and chemical composition of the crusts.

### Heterogeneity of microbial abundance on Fe–Mn crusts

The results of the Q-PCR analysis indicated that 16S rRNA gene copy abundance varied among crust samples. This may reflect the heterogeneity of microbial abundance on the sample surface within a crust sample, which is consistent with microscopic observations (Fig. 2). This heterogeneity in microbial abundance (ranging between 10^4^ and 10^9^ copies g^−1^) on the surface of seafloor basaltic rocks collected from various areas, such as the Arctic Ocean, Central Pacific, and southwest Pacific, has already been reported (15, 54, 64). Due to this heterogeneity, difficulties are associated with assessing the relationship between microbial abundance on the crust surface and water depth, similar to the case of basaltic rocks in the Arctic Ocean (15) and global deep-sea surface sediments (10).

### High archaeal proportion on Fe–Mn oxides

The results of the Q-PCR and amplicon sequencing analyses of 16S rRNA genes suggest that MGI archaea is a major component of the microbial communities on Fe–Mn oxide deposits from the bathyal to abyssal zones. Since the median 16S rRNA gene copy numbers for bacteria were greater than those for archaea (61), the ratio of archaeal cells in all prokaryotic cells may be higher than those reflected by the above Q-PCR results. Nitahara and co-workers (2017) already showed a high proportion of archaea (~89% of all prokaryotic 16S rRNA gene copies) in bathyal Fe–Mn crusts collected in the Takuyo-Daigo Seamount by Q-PCR. A high proportion of archaea (>50%) has also been reported in abyssal Fe–Mn nodules in the South Pacific Gyre (57) and the Clarion and Clipperton Zone (5) by Q-PCR. Furthermore, previous findings (5, 43, 57) indicated that MGI dominate in archaeal populations using a 16S rRNA gene sequencing analysis, which is consistent with the present results. In contrast, previous studies reported that the archaeal proportion generally accounts for less than 10% of the whole microbial community on young basaltic rocks, which were not covered with thick Fe–Mn oxides, near ridges or active volcanic seamounts (15, 54, 64). Therefore, the predominance of MGI appears to be somewhat related to the abundant Fe–Mn oxides of the outcrops. Further analyses, such as the isolation and physiological characterization of MGI, are needed to clarify this relationship.

### Factors causing differences in community composition and diversity

The present study examined the microbial abundance, diversity, and community structure of bathyal and abyssal Fe–Mn crusts, in addition to those of the surrounding seawater and sediments, using the same experimental procedures. This approach minimized the methodological biases associated with PCR-based comparative analyses of microbial communities. We observed differences in microbial community diversity and composition among the habitats, among Fe–Mn crust surfaces along the water depth, and among positions on each crust surface (*i.e.*, top and bottom sides) at population and individual OTU levels.

One factor causing the observed difference among habitats may be differences in substrates, *i.e.*, Fe and Mn oxides for the crust, silicate for the sediment, and liquid seawater, as previously reported (35, 43, 58). Variations in the lifestyles of free-living microbes and solid-surface attached microbes may cause differences between communities in solid substrates and seawater. Regarding the difference between the crusts and other habitats, abundant Fe–Mn oxides in the crust may change environmental characteristics, which directly affects microbial activity. Deep-sea environments are organic-poor; moreover, organic carbon compounds in the deep sea may be recalcitrant for microorganisms (26). Mn oxides are known to abiotically decompose recalcitrant organic carbon (*i.e.*, humic substances) to simple carbon compounds, such as pyruvate, acetaldehyde, and formaldehyde (63), which are easily used as energy/carbon sources by microorganisms. In addition, Mn oxides are known to catalyze the detoxification of hydrogen peroxide (H_2_O_2_) (2), which is present at low, but detectable concentrations (~1–10 nM) in deep oceans (70, 73, 74). H_2_O_2_ is a byproduct of aerobic respiration and a source of hydroxyl radicals, which damage microorganisms (24). α-Keto acids, such as pyruvate, which may be produced in a reaction between Mn oxides and humic substances (63), are also known to degrade H_2_O_2_ and facilitate the growth of ammonia oxidizers in MGI via H_2_O_2_ detoxification (31). Furthermore, since Mn oxides are toxic to some microorganisms, the tolerance of members of microbial communities to Mn oxides may result in the differences observed in community structures, as previously described (58). Thus, the abundance and reactivity of Mn oxides may cause the differences noted in microbial community structures between the crusts and other habitats.

Even among Fe–Mn crust samples, we observed differences in community compositions and diversities through the water depths. This result suggests that water depth is a major factor shaping microbial community structures on the crust, as previously reported for the deep-sea water column (52, 62, 71). The difference in DO through the water depths (Fig. S3) may be a factor shaping microbial communities on the crusts. In the shallower zones (1,200–1,400 m depth) with low DO (only 25% of the saturated concentration), only microbes adapted to low DO appeared to grow well. *Chromatiales*, which was abundantly detected in shallower samples (Fig. 6A), included microaerophiles (59). Accordingly, members of other taxonomic groups, such as *Nitrospirae* and *Acidiferrobacterales*, which were abundantly detected in shallower samples (Fig. 6A), may include microaerophiles. In addition to DO, differences in water depths reflect those in many physicochemical factors, such as pressure, salinity, temperature, and nutrient availability (21, 45) (Fig. S3). The concentrations of elements (such as Al, Co, Fe, and Mn) in the crusts also changed depending on the water depth in the North Pacific (69). Further analyses are needed to clarify whether and how these physicochemical factors are associated with the differences observed in microbial communities.

The present results showed differences in community compositions that depended on the position on the Fe–Mn crust surface within a sample. A potential factor causing these differences may be the availability of sinking particles with organic matter. Sinking particles are easily sedimented on the top side, but not on the bottom side. Grayish sandy sediments were only observed on the top side (Fig. S1). Thus, the availability of organic matter supplied from the particles may be less on the bottom side. This may explain the result showing that *Nitrosomonadales*, including autotrophic ammonia-oxidizing bacteria, at the population level for deeper samples (Fig. 6D) and some OTUs of MGI, including autotrophic ammonia-oxidizing archaea, at the individual OTU level for shallower and deeper samples (Fig. 7), were more abundant on the bottom side than on the top side.

## Conclusion

The present study showed the microbial localization, abundance, and community structures of Fe–Mn crusts, in addition to the surrounding sediments and bottom seawater as references, in bathyal and abyssal zones at the Takuyo-Daigo Seamount using comprehensive culture-independent analyses, including correlative light and electron microscopies, PCR amplicon sequencing, and Q-PCR of 16S rRNA genes. Further functional characterizations of these microbial communities (such as comparative metagenomics/metatranscriptomics/metaproteomics, and the cultivation and physiological characterization of each microorganism) and detailed measurements of environmental properties (such as the types of components and concentrations of organics) targeting more seamounts are needed to comprehensively understand the commonality and variability of microbial communities on outcrops in deep oceans, their contribution and mechanisms of Fe–Mn crust formation, and, ultimately, their ecological significance in the global ocean.

## Supplementary information
