# Supplementary material for: Heterogeneity of Microbial Communities on Deep-Sea Ferromanganese Crusts in the Takuyo-Daigo Seamount
Source: Microbes Environ. 2018 Oct 30;33(4):366–77. doi: 10.1264/jsme2.ME18090 (PMC6307992; doi:10.1264/jsme2.ME18090)
Supplement: Supplementary file 1 [file 33_366_s1.pdf]

Supplementary information

Heterogeneity of microbial communities on deep-sea ferromanganese crusts in the Takuyo-Daigo Seamount

S Kato*, T Okumura, K Uematsu, M Hirai, K Iijima, A Usui, K Suzuki

*Corresponding author,
Shingo Kato, Ph.D.
Ore Genesis Research Unit, Project Team for Development of New-generation Research Protocol for Submarine Resources, JAMSTEC
2-15 Natsushima, Yokosuka, Kanagawa, 237-0061 Japan
E-mail: skato@jamstec.go.jp

Supplementary text

Large filamentous organisms (> 10 μm in width, > 1 mm in length in some cases) were observed on the surface of some crust samples by SEM (Fig. S5A, D, E, and K). An early microscopic study of ferromanganese nodules demonstrated the presence of such filaments on the nodule surface (Burnett and Nealson, 1981). In the present study, SEM-EDS analysis showed that the filaments contained carbon (C) and iodine (I). The peak for I L-β at 4.22 keV was detected on the filaments, but not on the other surfaces (Fig. S7A and E). The large filaments potentially take up iodide from seawater, like brown seaweeds (Küpper et al., 1998). A peak for I L-α at 3.94 keV was also detected on the filaments; however, based on the energy resolution of EDS, the I L-α peak was difficult to distinguish from the Ca K-β peak at 4.01 keV. SEM also revealed the presence of trenches in the interior of the crusts, with a size and morphology corresponding to the large filaments (Fig. S5F and I). The trenches may be the trace of large filaments buried in the crusts.

Supplementary references

Burnett, B.R., and Nealson, K.H. (1981) Organic films and microorganisms associated with manganese nodules. *Deep Sea Research Part A Oceanographic Research Papers*, 28: 637-645.

Küpper, F.C., Schweigert, N., Ar Gall, E., Legendre, J.M., Vilter, H., and Kloareg, B. (1998) Iodine uptake in laminariales involves extracellular, haloperoxidase-mediated oxidation of iodide. *Planta*, 207: 163-171.

Supplementary table legends

Table S1. Sample list with the amount of extracted DNA, Q-PCR, and amplicon sequencing results.

Table S2. Alpha diversity measurements.

Supplementary figure legends

Fig. S1. On-site photos of the sampling areas in the Takuyo-Daigo Seamount. Crusts on the exposed rocks were observed at every dive ranging from 1150 m to 5500 m. In some cases, whitish sandy sediments slightly covered the crusts.

Fig. S2. Map of the Takuyo-Daigo Seamount. (A) Location of the Takuyo-Daigo Seamount. (B) Bathymetric map of the Takuyo-Daigo Seamount. Sampling points on the southern ridge and the flat-top of the seamount are indicated by red dots.

Fig. S3. Water depth profile of temperature, salinity, and dissolved oxygen concentration. The dissolved oxygen concentration at the sea surface was as 100% (corresponding to approximately 200 μM under the conditions).

Fig. S4. Photos of the crust samples used in this study. Top surface (left panels), bottom surface (middle panels), and section surface (right panels) of the crusts are shown. Blackish Fe-Mn oxides covered the basement rocks. Subsamples shown by red arrows and ID were collected from each sample and used for the following analyses. The bottom surface of some samples (679R1 and 684R1) were attached to the seafloor sediments, and clay-like materials (CyB12 and CyB45) were observed on the attached surface. Grayish white sandy sediments were slightly covered on the up-side surface of some samples (678R1, 679R1, 682R2, 682R3, 683R7) indicated by sky-blue arrows for 678R1 and 679R1.

Fig. S5. Scanning electron microscopic images of the crust samples. The magnification of the images is low to high from the left to right panels. Red boxes indicate the view area for the righter panels. Images of the samples (A–C) 678R1, (D–F) 679R1, (G) 680R2, (H and I) 682R2, (J) 682R3, and (K–M) 684R1 are shown. (A, B, D, G, H J, K, and L) Top surface of the crusts appeared bumpy. Cocci- and rod-shaped cells and nano-sized wire-like structures were observed at high magnification (> ×20,000). (lower panels of H and L) Diatoms were accumulated in the valleys. (A, D, E, and the lower panels of K) Large filaments on the top surface. (C, F, I, and M) Crashed surface (removed bumpy parts), i.e., inside of the crusts. Although no apparent cells were observed, cell-sized hollows (indicated by red arrows) and

trenches (indicated by blue arrows) were observed. The hollows and trenches likely originated from microbial cells and large filaments, respectively.

Fig. S6. Representative result of correlative light and scanning electron microscopy with EDS mapping of a valley on the top-side surface of the sample 679R1. As in the images of 684R1 in Fig. 3, the counts for Mn were low in the valley, whereas the counts for Fe were comparable to the surrounding Fe-Mn crusts. Spotty Si and Al detections likely originated from diatoms and sedimentary debris.

Fig. S7. Representative results of energy-dispersive X-ray spectroscopy (EDS). (A and B) Scanning electron microscopic images with EDS results of the sample 679R1. (A) Large filaments and (B) diatom-like debris on the top-side of the crust surface. The same view for the Fig. S5D. The peaks for carbon and iodine at the analyzed points (001,003,004) for filaments were higher than those in surrounding crusts (005,006,008). (C–E) The results of sample 684R1. The same view for the Fig. S5K and L. Results of a (C) hill and (D) valley on the top-side surface of crusts. In the EDS results, the peaks for Si and Ca were highly observed at the analyzed points for the valley, which likely originated from diatoms with silicate core and from foraminifera composed of calcium carbonate. (E) A large filament. As well as the (A), the peaks of carbon and iodine were detected. For all EDS mapping results, the peaks of Os were derived from the coating elements.

Fig. S8. Water depth profile of the prokaryotic and archaeal 16S rRNA gene copy numbers and the ratio of archaeal 16S rRNA gene copy numbers to the total prokaryotic 16S rRNA gene copy numbers. Blue, orange, and gray dots indicate the results of top-side (MnT), bottom-side (MnB), and shallower sediments (SedS), respectively. Error bars indicate standard deviation of the mean.

Fig. S9. Alpha diversity of the samples based on habitat types and positions. (A) Chao1 species richness estimates, Shannon diversity index, and inverse of Simpson diversity index. Water depth of each points are represented by color gradation (from blue to black, as from shallow to deep). (B) The *p* values of Wilcoxon rank sum test between each data of alpha diversity plotted in Fig. S9A. Yellow cells, $p < 0.05$. Red, $p < 0.01$. (C) Rarefaction curves of (sub)samples in the 16S rRNA gene amplicon tag sequencing analysis.

Fig. S10. Relative abundance of taxa showing higher relative abundance (> 2.5%) in each sample. (A) Bar graph showing the relative abundance of taxa at phylum or class level (represented by colors indicated in the right box) for all samples. (B) Box plot of the relative abundance of taxa for the habitat type (represented by colors indicated in the bottom-right box). Relative abundance of taxa at order level for (C and D) Marine Group I of Thaumarchaeota, (E and F) Alphaproteobacteria, and (G and H) Gammaproteobacteria. (C, E, and G) Bar graph showing the relative abundance of taxa (represented by colors indicated

in the bottom boxes) for all samples. (D, F, and H) Box plot of the relative abundance of taxa for the habitat types (represented by colors indicated in the top boxes).

Fig. S11. Phylogenetic tree of OTUs affiliated with Marine Group I of Thaumarchaeota. The OTU IDs in orange and green were detected in the PCR amplicon tags and the MAG MnTg01, respectively. Each name of clade was defined based on cultivated species and previous reports. The scale bar represents 0.1 nucleotide substitutions per sequence position. Bootstrap values (> 50% of 1000 replicates) are indicated at nodes.

Fig. S12. Relative abundance of taxa at the phylum or class level, showing the relative abundance of (A and B) 0.1–2.5% and (C and D) < 0.1% in each sample. (A and C) Bar graph showing the relative abundance of taxa (represented by colors indicated in the right box) for all samples. (B and D) Box plot of the relative abundance of taxa for the habitat types (represented by colors indicated in the bottom-right box).

Fig. S13. Comparison of relative abundance of representative taxa among positions and water depths. Box plot of the relative abundance for the taxa at phylum or class level with (A) high abundance (≥ 2.5%), and (B) low (< 2.5%) abundance is shown. (C) Box plot for taxa at order level for Alphaproteobacteria, Betaproteobacteria, Deltaproteobacteria, Gammaproteobacteria, and Marine Group I of Thaumarchaeota. The positions and water depths are represented by colors indicated in the top boxes.

Fig. S14. Box plot of relative abundance of core and abundant OTUs for the Fe-Mn crusts. The taxonomic affiliations are indicated at the left of the OTU IDs. The results from the crusts, sediments, and seawater are represented by blue, orange, and green, respectively. The OTUs (Otu512 and Otu520) with asterisks showed significantly different abundance between the top- and bottom-side of the crusts (Fig. 7; see text for details).

Fig. S15. Shared and unique OTUs among habitats. (A) Venn diagram showing the number of the unique and shared OTUs. (B) Rank abundance plots of the number of detected samples for crust-unique OTUs. Taxonomic affiliation of unique OTUs for each habitat (C) at the phylum and class level and (D) at the order level for Gammaproteobacteria, Deltaproteobacteria, and Alphaproteobacteria.

Fig. S16. Venn diagram showing the number of unique and shared OTUs among the positions of the crust surface with different water depths.

Table S1. Sample list with the amount of extracted DNA, Q-PCR, and tag sequencing results

Sample name	Sampling date	Dive#	Latitude	Longitude	Water Depth (m)	Subsample ID	Sublocation	Substrate	DNA amount (ng/g or L sample)	16S rRNA gene copy numbers (copies/g)					16S amplicon sequencing
										Univeral	Uni-std	Archaeal	Arc-std	Arc/Uni	
Mn crust and clay															
678R1	2016/1/12	678	22:44.606N	153:15.978E	1432	MnT14a	Top	Fe-Mn oxide	111	1.9.E+07	2.7.E+06	3.1.E+06	5.1.E+05	16.2%	Detected
						MnT14b	Top	Fe-Mn oxide	117	8.7.E+06	3.6.E+05	1.5.E+06	1.6.E+05	17.6%	Detected
						MnB14a	Bottom	Fe-Mn oxide	215	7.2.E+07	9.2.E+06	1.1.E+07	2.2.E+06	15.2%	Detected
						MnB14b	Bottom	Fe-Mn oxide	107	2.6.E+07	3.1.E+06	7.3.E+06	1.7.E+06	28.5%	Detected
679R1	2016/1/13	679	22:51.104N	153:26.973E	1154	MnT12a	Top	Fe-Mn oxide	91.2	1.9.E+07	2.5.E+06	1.7.E+06	4.2.E+05	9.2%	Detected
						MnT12b	Top	Fe-Mn oxide	66.6	1.1.E+07	8.5.E+05	3.6.E+06	5.9.E+05	32.3%	Detected
						RkI12a	Inside	Rock	N.D.	N.D.	N.D.	N.D.	N.D.	N.A.	N.D.
						RkI12b	Inside	Rock	N.D.	3.4.E+04	7.2.E+03	N.D.	N.D.	N.A.	N.D.
						CyB12	Bottom	Clay	161	3.6.E+07	1.4.E+06	1.1.E+07	2.5.E+06	30.7%	Detected
680R2	2016/1/14	680	22:30.722N	153:12.983E	5517	MnT55a	Top	Fe-Mn oxide	76.4	2.2.E+07	2.1.E+06	3.9.E+06	2.5.E+05	18.1%	Detected
						MnT55b	Top	Fe-Mn oxide	75.7	8.9.E+06	4.2.E+05	4.0.E+06	5.2.E+05	45.3%	Detected
						MnB55a	Bottom	Fe-Mn oxide	120	3.4.E+07	4.1.E+06	6.6.E+06	7.8.E+05	19.1%	Detected
						MnB55b	Bottom	Fe-Mn oxide	84.2	1.2.E+07	2.9.E+05	5.6.E+06	7.2.E+05	45.0%	Detected
682R2	2016/1/16	682	22:40.853N	153:14.364E	2988	MnT30Aa	Top	Fe-Mn oxide	11.7	9.6.E+05	8.8.E+04	2.2.E+05	1.3.E+05	22.6%	N.D.
						MnT30Ab	Top	Fe-Mn oxide	34.8	5.4.E+06	3.9.E+05	2.8.E+06	6.3.E+05	52.5%	Detected
						MnI30Aa	Inside	Fe-Mn oxide	10.6	1.5.E+06	3.3.E+05	2.9.E+05	2.0.E+04	18.6%	N.D.
						MnI30Ab	Inside	Fe-Mn oxide	N.D.	6.0.E+05	8.2.E+04	3.1.E+05	4.2.E+04	51.5%	N.D.
682R3	2016/1/16	682	22:40.853N	153:14.361E	2987	MnT30Ba	Top	Fe-Mn oxide	51.3	9.5.E+06	7.8.E+05	2.6.E+06	1.5.E+06	27.3%	Detected
						MnT30Bb	Top	Fe-Mn oxide	57.7	4.6.E+06	2.4.E+05	2.2.E+06	4.0.E+05	47.9%	Detected
						MnT30Bc	Top	Fe-Mn oxide	41.1	9.9.E+06	1.7.E+06	1.6.E+06	2.0.E+05	16.4%	Detected
						MnB30Ba	Bottom	Fe-Mn oxide	39.0	9.4.E+06	1.1.E+06	1.9.E+06	1.6.E+05	20.5%	Detected
						MnB30Bb	Bottom	Fe-Mn oxide	30.3	4.5.E+06	1.7.E+05	2.7.E+06	6.7.E+05	59.1%	Detected
						MnI30Ba	Inside	Fe-Mn oxide	N.D.	N.D.	N.D.	N.D.	N.D.	N.A.	N.D.
						MnI30Bb	Inside	Fe-Mn oxide	N.D.	1.9.E+05	1.1.E+04	1.6.E+05	4.8.E+03	85.6%	N.D.
683R7	2016/1/18	683	22:29.192N	153:12.069E	5373	MnT54a	Top	Fe-Mn oxide	17.5	3.4.E+06	3.4.E+05	7.1.E+05	1.6.E+05	20.9%	Detected
						MnT54b	Top	Fe-Mn oxide	78.6	6.0.E+06	2.6.E+05	3.0.E+06	3.7.E+05	49.5%	Detected
						MnI54a	Inside	Fe-Mn oxide	N.D.	N.D.	N.D.	N.D.	N.D.	N.A.	N.D.
						MnI54b	Inside	Fe-Mn oxide	N.D.	N.D.	N.D.	N.D.	N.D.	N.A.	N.D.
684R1	2016/1/19	684	22:37.474N	153:12.915E	4480	MnT45a	Top	Fe-Mn oxide	244	5.8.E+07	5.4.E+06	8.1.E+06	1.2.E+06	14.0%	Detected
						MnT45b	Top	Fe-Mn oxide	158	2.7.E+07	1.8.E+06	1.1.E+07	2.6.E+06	40.0%	Detected
						MnB45a	Bottom	Fe-Mn oxide	40.2	3.6.E+06	5.3.E+05	9.5.E+05	2.4.E+05	26.3%	Detected
						MnB45b	Bottom	Fe-Mn oxide	40.7	5.6.E+06	2.8.E+05	4.0.E+06	5.0.E+05	71.2%	Detected
						MnI45a	Inside	Fe-Mn oxide	9.53	8.7.E+05	2.2.E+05	9.2.E+04	5.3.E+04	10.5%	N.D.
						MnI45b	Inside	Fe-Mn oxide	N.D.	4.3.E+05	4.5.E+04	2.5.E+05	2.5.E+04	58.9%	N.D.
						CyB45a	Bottom	Clay	N.D.	3.8.E+05	2.7.E+04	1.1.E+05	9.8.E+03	28.8%	N.D.
						CyB45b	Bottom	Clay	15.5	2.2.E+06	2.0.E+05	2.5.E+06	2.2.E+05	111.9%	Detected
Sediment															
678C1	2016/1/12	678	22:44.603N	153:15.971E	1432	SedS14	Upper	Sediment	290	4.6.E+07	2.1.E+06	2.1.E+07	7.1.E+05	44.7%	Detected
679C1	2016/1/13	679	22:51.100N	153:26.973E	1154	SedS12	Upper	Sediment	579	1.2.E+08	4.4.E+06	3.1.E+07	1.0.E+06	26.4%	Detected
680C1	2016/1/14	680	22:30.570N	153:13.089E	5577	SedS56	Upper	Sediment	240	5.8.E+07	2.7.E+06	2.6.E+07	2.6.E+06	44.9%	Detected
						SedD56	Lower	Sediment	15.8	1.3.E+06	8.6.E+04	8.4.E+05	2.0.E+05	64.7%	Detected
684C1	2016/1/19	684	22:37.509N	153:13.089E	4374	SedS44	Upper	Sediment	182	4.7.E+07	2.1.E+06	2.6.E+07	2.4.E+06	55.5%	Detected
						SedD44	Lower	Sediment	4.41	2.3.E+05	4.9.E+04	8.3.E+04	2.8.E+03	35.3%	Detected
Seawater															
678W1	2016/1/12	678	22:44.606N	153:15.956E	1426	Asw14A	-	Seawater	5.50	1.0.E+02	2.1.E+01	2.9.E+01	4.9.E+00	28.3%	Detected
678W2			22:44.623N	153:15.962E	1385	Asw14B	-	Seawater	5.64	1.6.E+02	1.4.E+01	6.1.E+01	4.9.E+00	39.2%	Detected
679W1	2016/1/13	679	22:51.110N	153:26.961E	1150	Asw12	-	Seawater	2.97	3.9.E+01	1.3.E+00	1.2.E+01	1.7.E+00	31.1%	Detected
680W1	2016/1/14	680	22:30.555N	153:13.219E	5582	Asw56	-	Seawater	N.D.	N.D.	N.D.	N.D.	N.D.	N.D.	Detected
682W1	2016/1/16	682	22:40.857N	153:14.363E	2986	Asw30	-	Seawater	N.D.	N.D.	N.D.	N.D.	N.D.	N.D.	Detected
684W1	2016/1/19	684	22:37.454N	153:12.892E	4479	Asw45	-	Seawater	29.0	2.2.E+03	3.9.E+02	9.6.E+01	9.9.E+00	4.3%	Detected

N.D., not detected; N.A., not applicable

Table S2. Alpha diversity measurements

Subsample ID	Number of reads after filtering	Observed OTUs	Chao1 species estimates (S.E.)	Shannon diversity index	Inverted Simpson diversity index
Mn crust					
MnT12a	52468	2463	3480 (91)	5.39	60.32
MnT12b	41444	2216	3215 (91)	5.26	45.36
MnT14a	25675	1886	3132 (114)	5.19	49.45
MnT14b	60348	2810	4019 (99)	5.33	55.31
MnB14a	57121	2480	3664 (101)	5.10	38.26
MnB14b	41708	2465	3762 (110)	5.32	35.18
MnT30Ab	43941	2892	3764 (74)	5.58	45.16
MnT30Ba	44347	2349	2954 (60)	5.40	55.75
MnT30Bb	61697	3078	3899 (71)	5.62	57.76
MnT30Bc	41211	2120	2529 (44)	5.15	43.67
MnB30Ba	46999	2402	3163 (73)	5.50	63.25
MnB30Bb	53165	2522	3313 (74)	5.46	56.04
MnT45a	36365	2088	3211 (100)	4.91	33.00
MnT45b	58609	2549	3687 (96)	4.84	28.76
MnB45a	39845	2420	3291 (80)	5.50	54.17
MnB45b	64514	2763	3496 (66)	5.42	54.93
MnT54a	22267	1694	2483 (79)	4.54	14.22
MnT54b	48545	2711	4243 (121)	5.18	34.78
MnT55a	37348	1875	3139 (119)	4.83	32.38
MnT55b	59609	1722	2892 (113)	3.73	10.89
MnB55a	40891	2161	3706 (134)	4.97	33.36
MnB55b	36597	1798	3335 (147)	4.69	29.06
Fe-Mn crust data combined (n=22)	*1014714*	*9441*	*9715 (26)*	*5.73*	*65.48*
Clay					
CyB12	52425	3118	4348 (97)	5.78	83.81
CyB45b	41060	2666	3553 (81)	5.95	89.17
Clay data combined (n=2)	*93485*	*4660*	*6018 (96)*	*6.20*	*120.66*
Sediment					
SedS12	63007	3989	5563 (110)	6.15	114.68
SedS14	75262	4329	5783 (99)	6.02	89.97
SedS44	72910	4006	5541 (109)	6.09	113.96
SedD44	73712	3814	5505 (118)	5.90	86.97
SedS56	72622	4031	5756 (119)	5.98	90.98
SedD56	63206	3310	4703 (107)	5.96	119.55
Sediment data combined (n=6)	*420719*	*7606*	*8620 (68)*	*6.21*	*113.36*
Seawater					
Asw12	44959	3169	4350 (93)	6.04	98.55
Asw14A	32311	2813	4415 (128)	5.97	88.00
Asw14B	48370	2957	4512 (123)	5.67	69.34
Asw30	68102	3046	4439 (108)	4.57	10.04
Asw45	82173	3267	4677 (105)	4.58	16.10
Asw56	38768	2691	4099 (115)	5.86	90.51
Seawater data combined (n=6)	*314683*	*6417*	*7593 (80)*	*5.64*	*44.28*

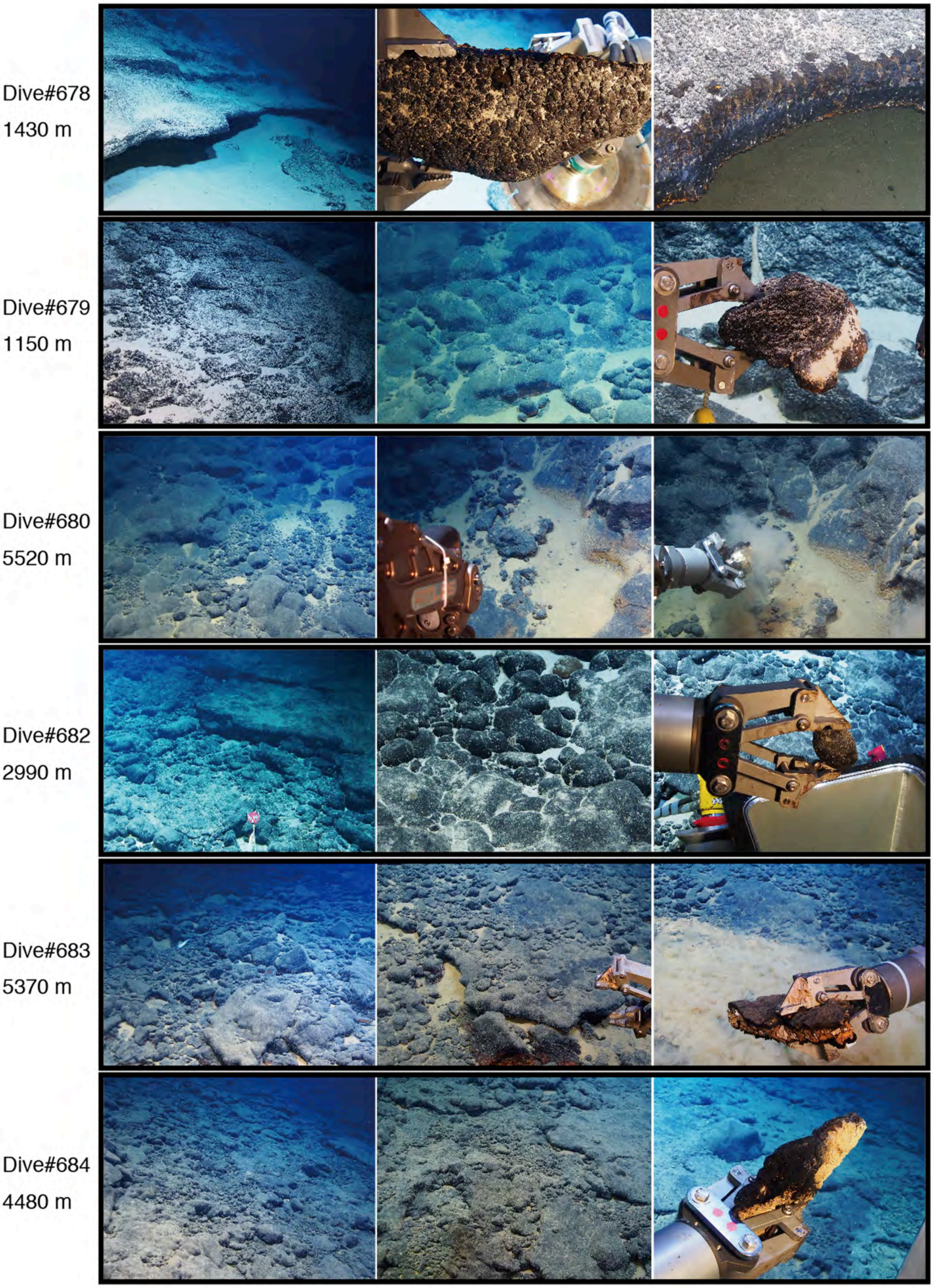
Dive#678
1430 m
Dive#679
1150 m
Dive#680
5520 m
Dive#682
2990 m
Dive#683
5370 m
Dive#684
4480 m

Fig. S1

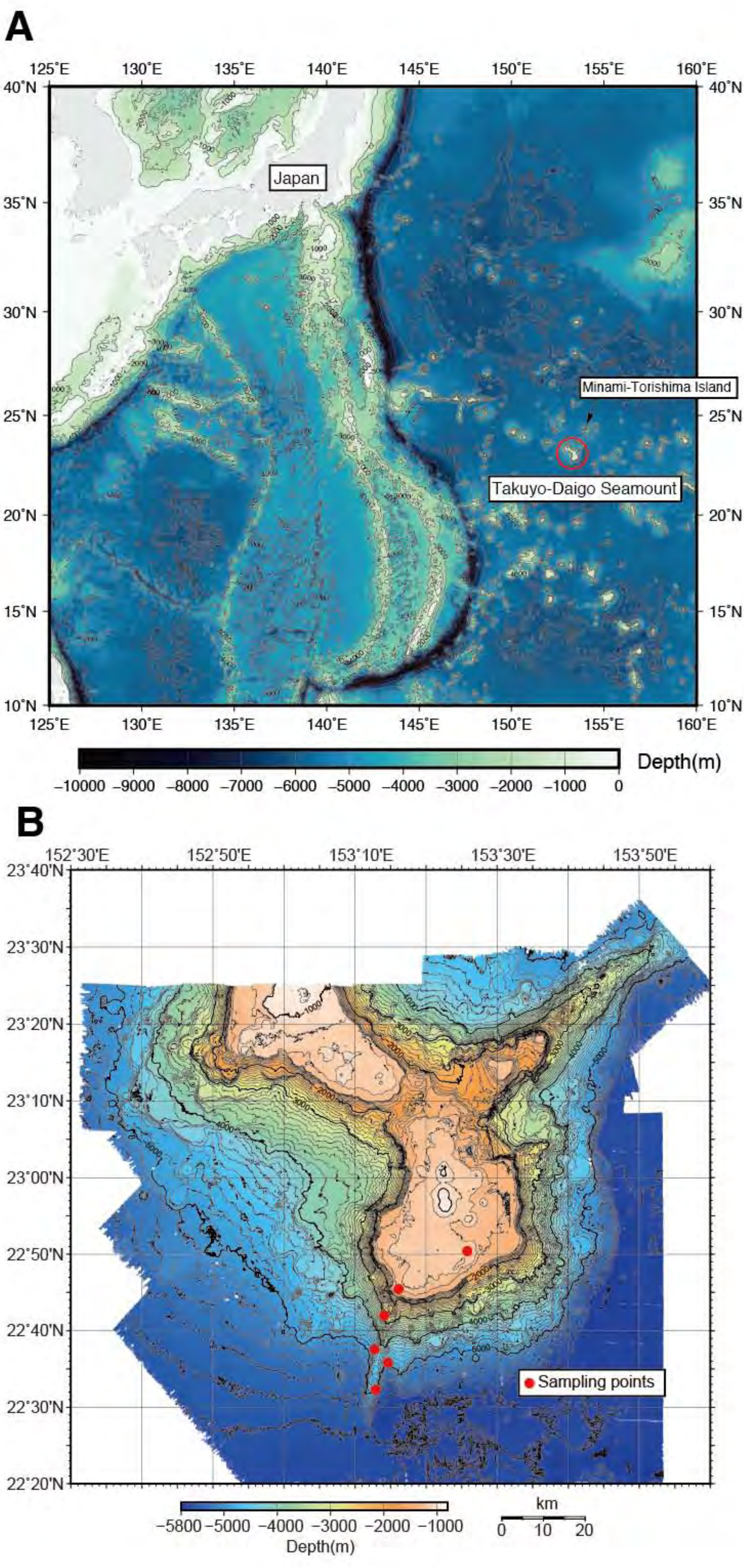
A
Japan
Minami-Torishima Island
Takuyo-Daigo Seamount
Depth(m)
B
Sampling points
Depth(m)
km

Fig. S2

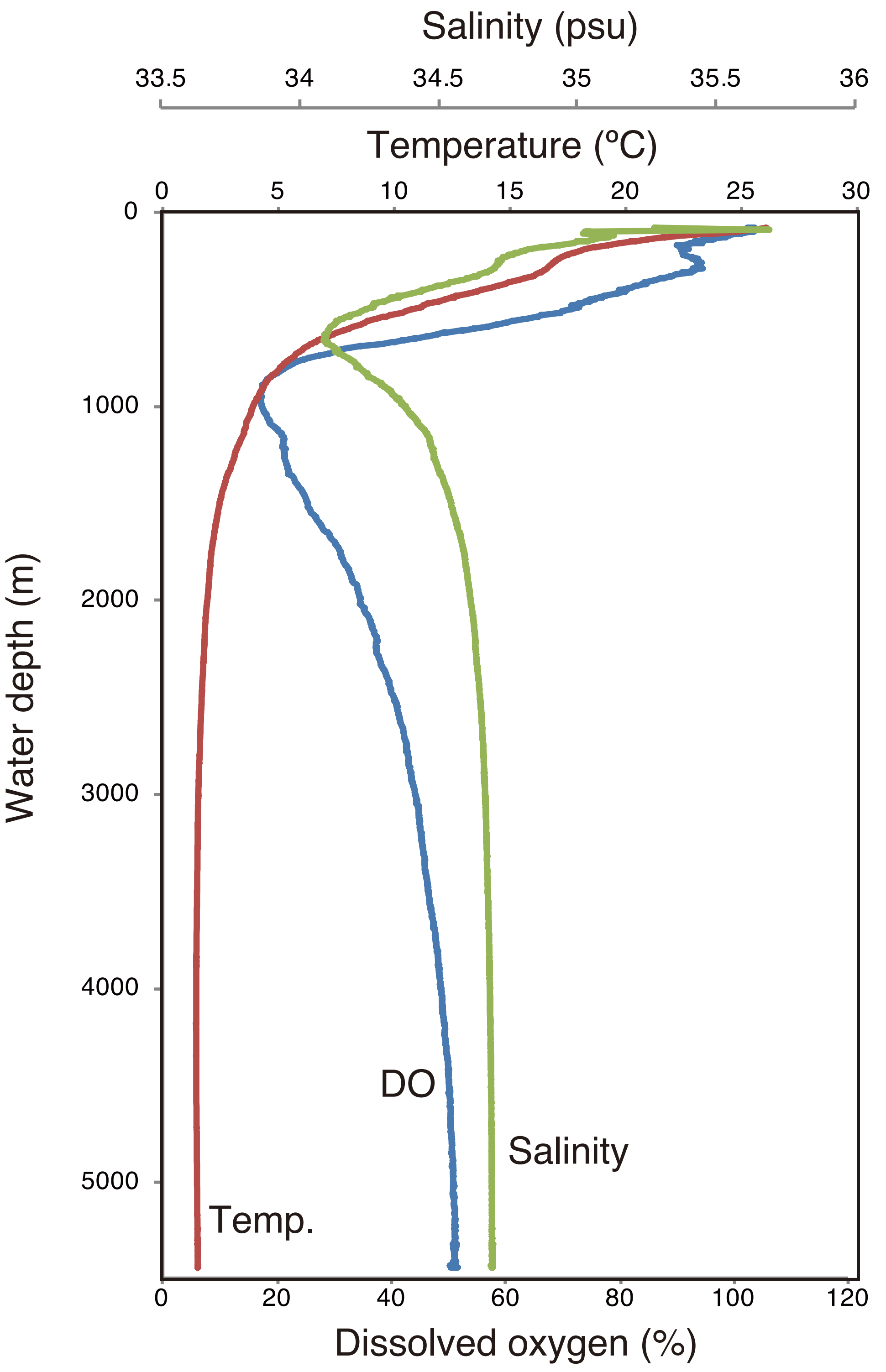
Salinity (psu)
33.5
34
34.5
35
35.5
36
Temperature (ºC)
0
5
10
15
20
25
30
Water depth (m)
0
1000
2000
3000
4000
5000
DO
Salinity
Temp.
0
20
40
60
80
100
120
Dissolved oxygen (%)

Fig. S3

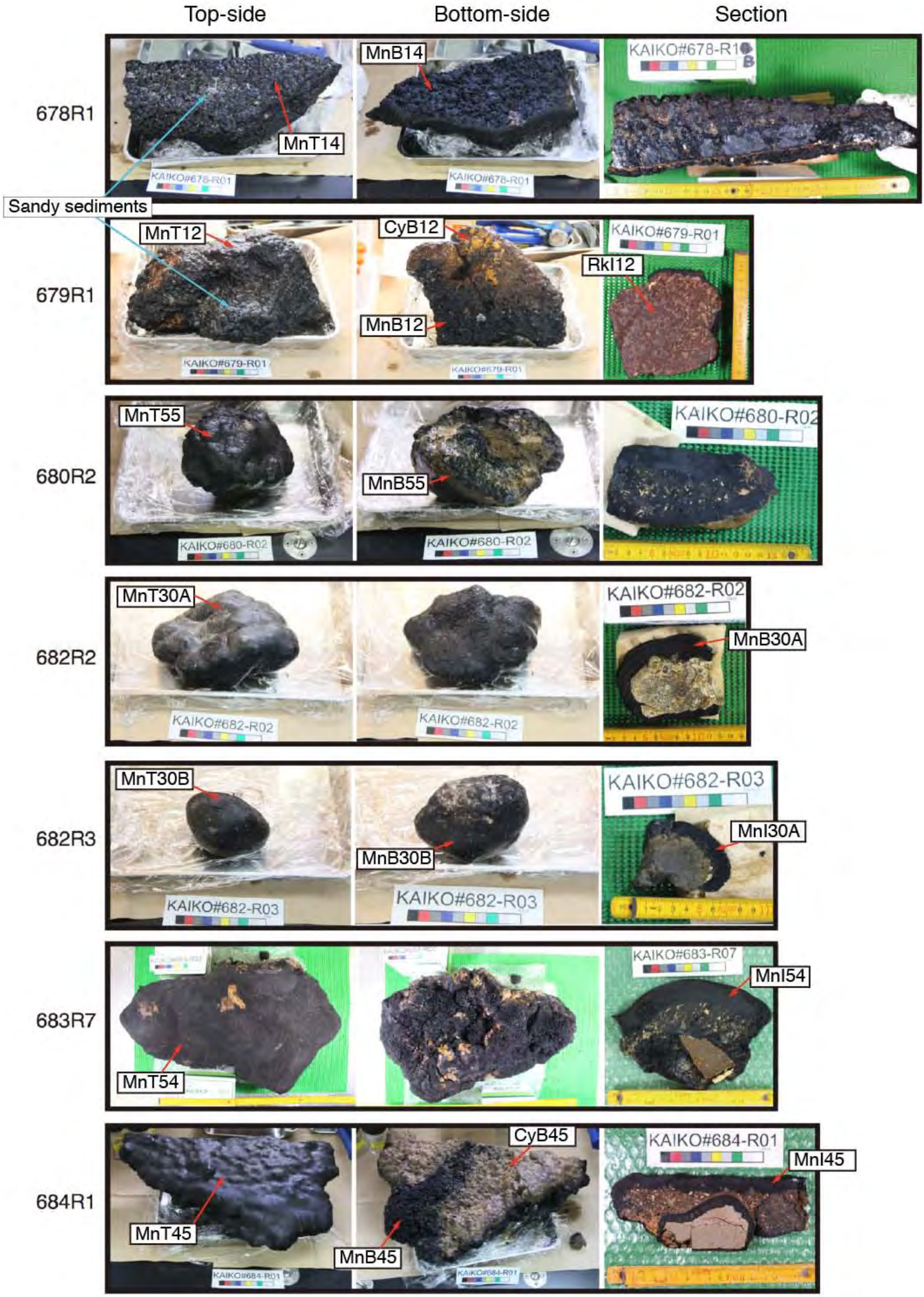
Top-side
Bottom-side
Section
678R1
679R1
680R2
682R2
682R3
683R7
684R1
Sandy sediments
MnT14
MnB14
KAIKO#678-R01
MnT12
CyB12
MnB12
RkI12
KAIKO#679-R01
MnT55
MnB55
KAIKO#680-R02
MnT30A
MnB30A
KAIKO#682-R02
MnT30B
MnB30B
MnI30A
KAIKO#682-R03
MnT54
MnI54
KAIKO#683-R07
MnT45
CyB45
MnB45
MnI45
KAIKO#684-R01

Fig. S4

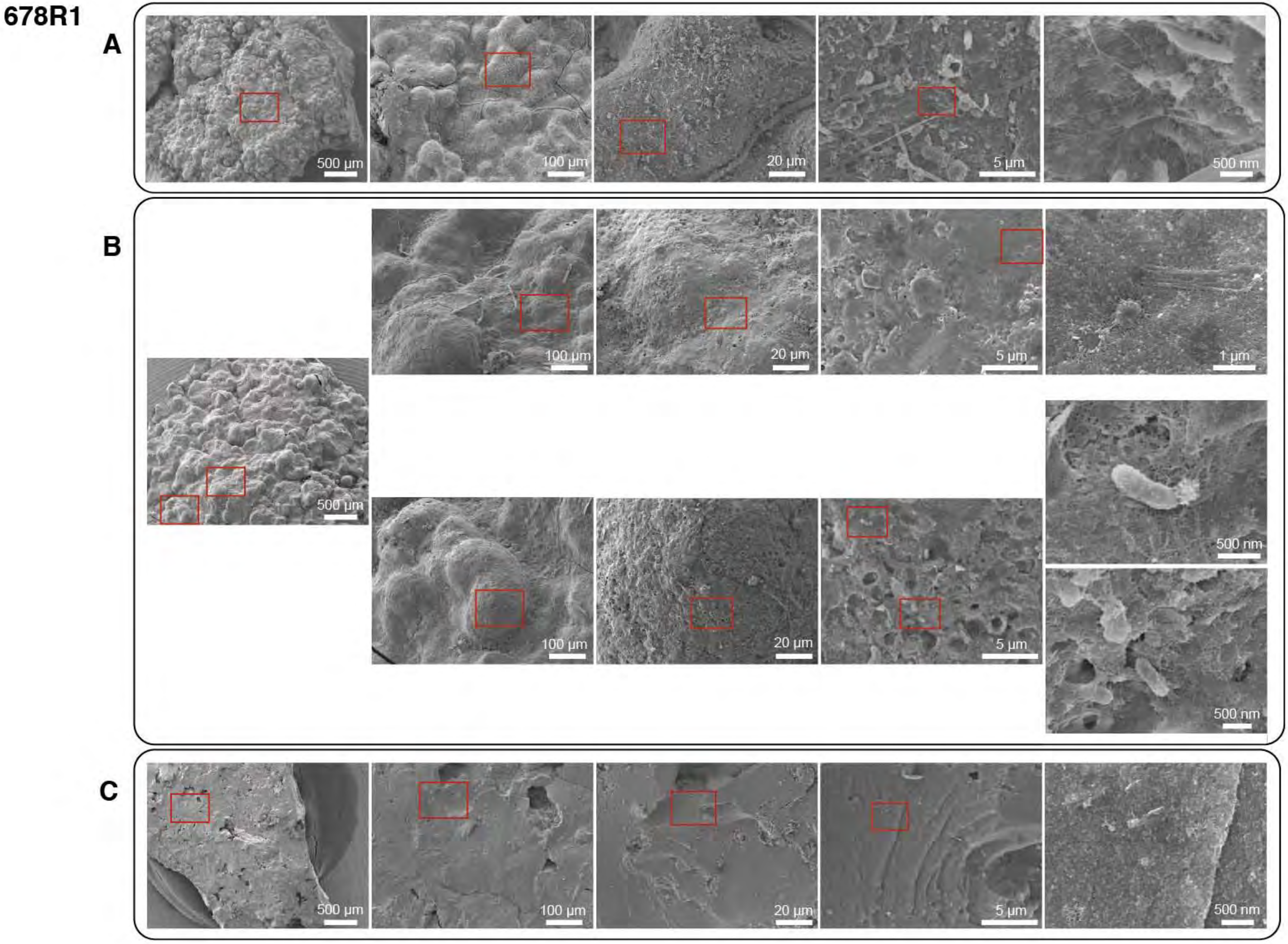
678R1
A
500 µm
100 µm
20 µm
5 µm
500 nm
B
100 µm
20 µm
5 µm
1 µm
500 µm
500 nm
100 µm
20 µm
5 µm
500 nm
C
500 µm
100 µm
20 µm
5 µm
500 nm

Fig. S5A-C

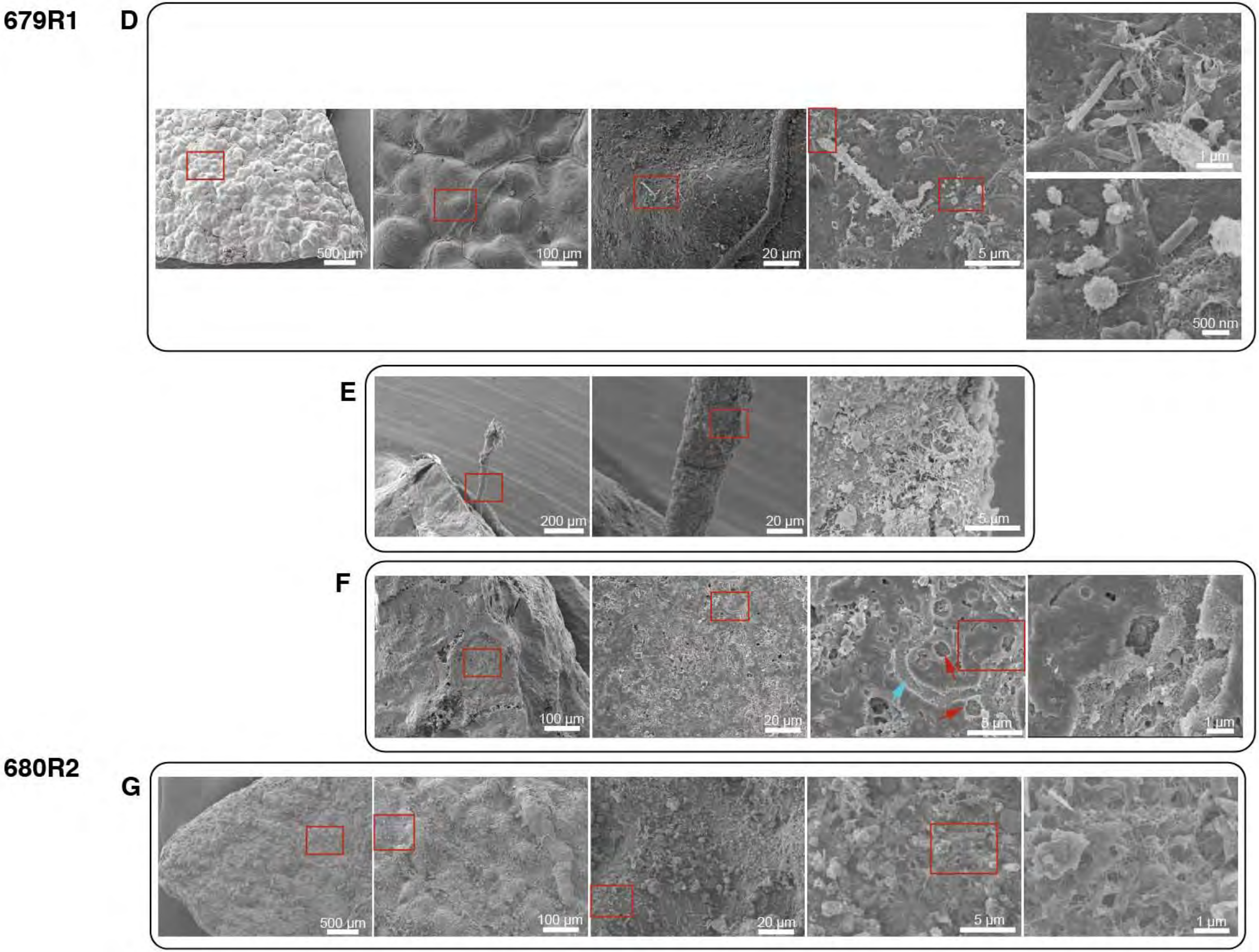
679R1
D
500 µm
100 µm
20 µm
5 µm
1 µm
500 nm
E
200 µm
20 µm
5 µm
F
100 µm
20 µm
5 µm
1 µm
680R2
G
500 µm
100 µm
20 µm
5 µm
1 µm

Fig. S5D-G

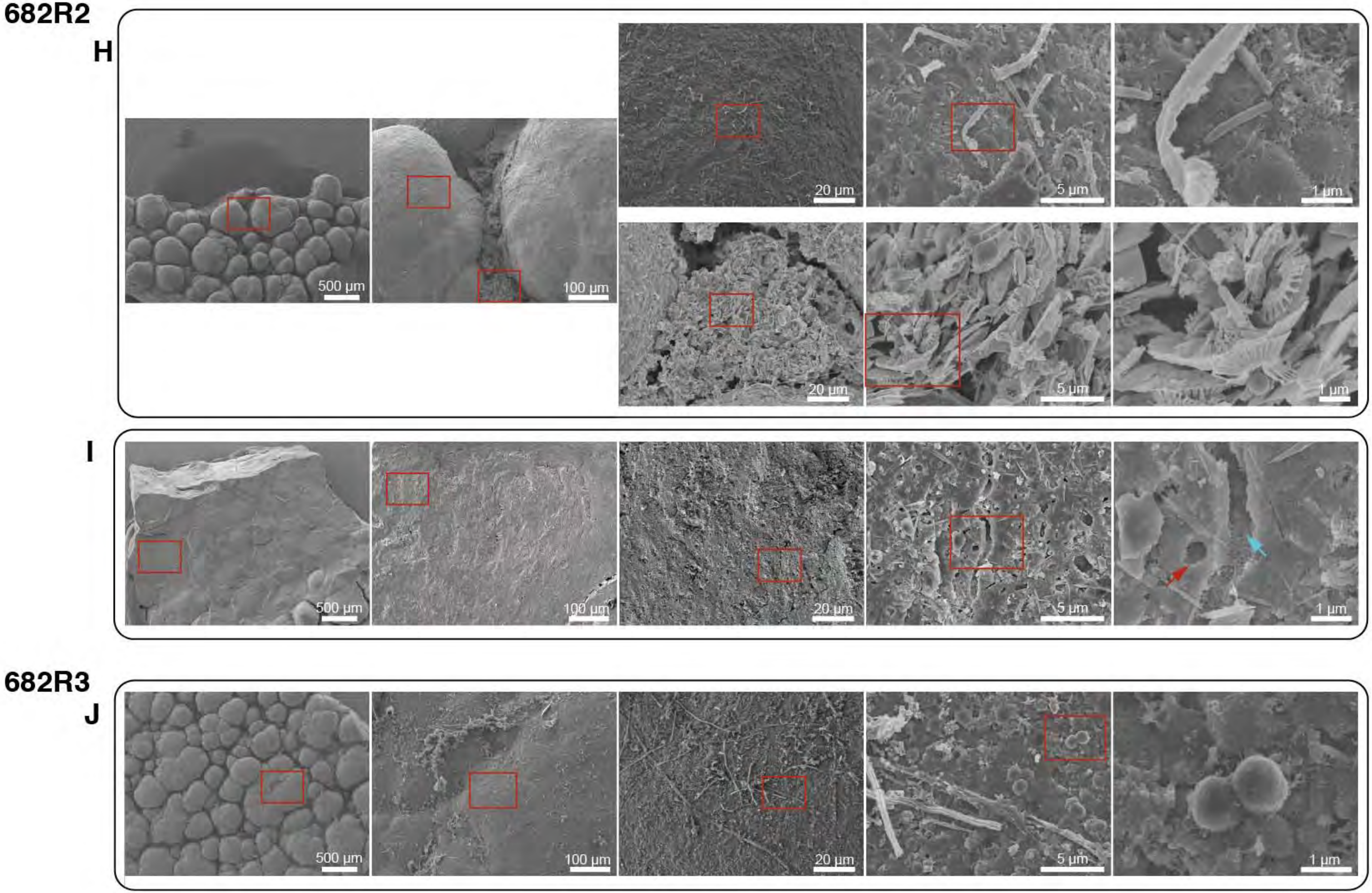
682R2
H
500 µm
100 µm
20 µm
5 µm
1 µm
20 µm
5 µm
1 µm
I
500 µm
100 µm
20 µm
5 µm
1 µm
682R3
J
500 µm
100 µm
20 µm
5 µm
1 µm

Fig. S5H-J

684R1

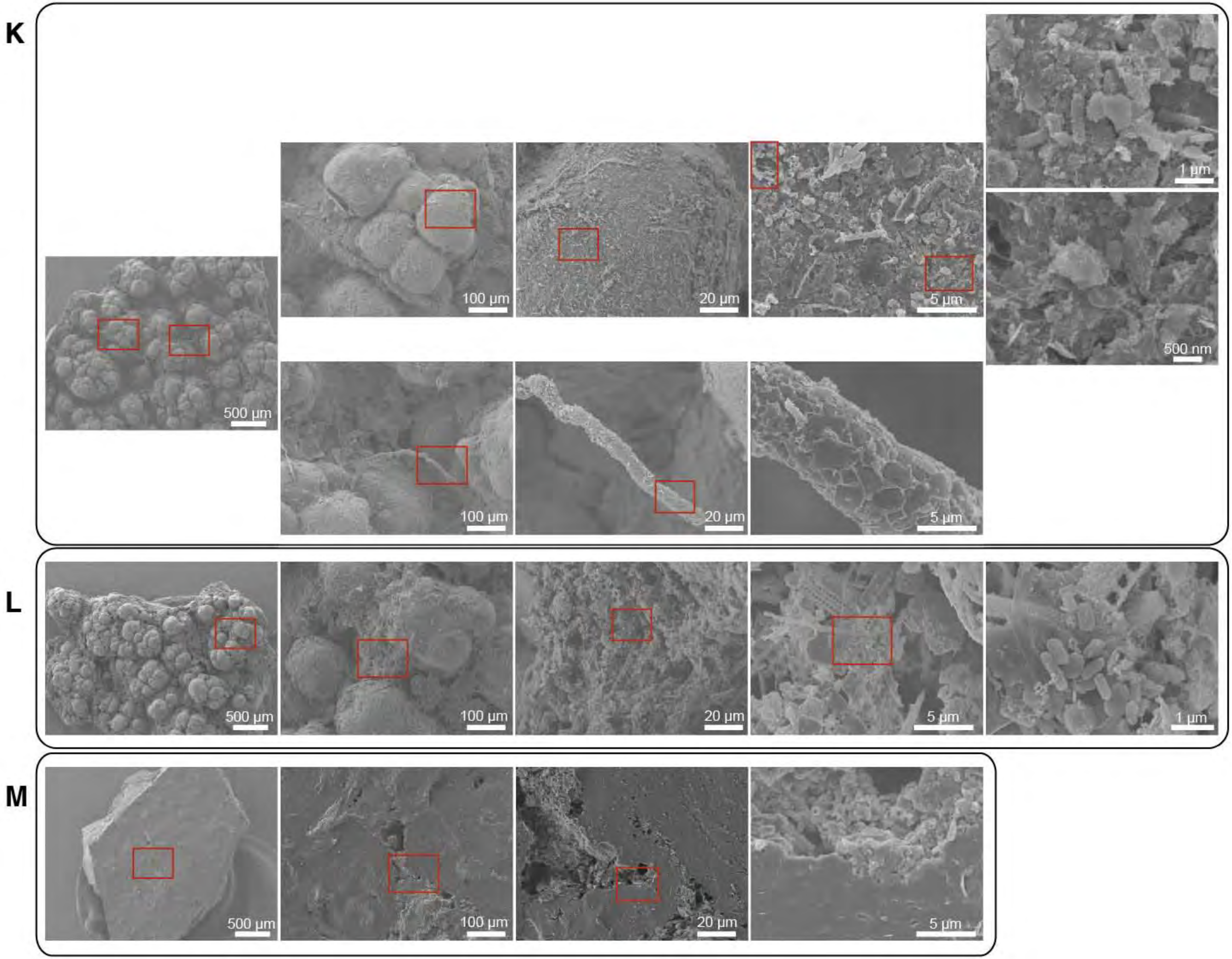

Fig. S5K-M

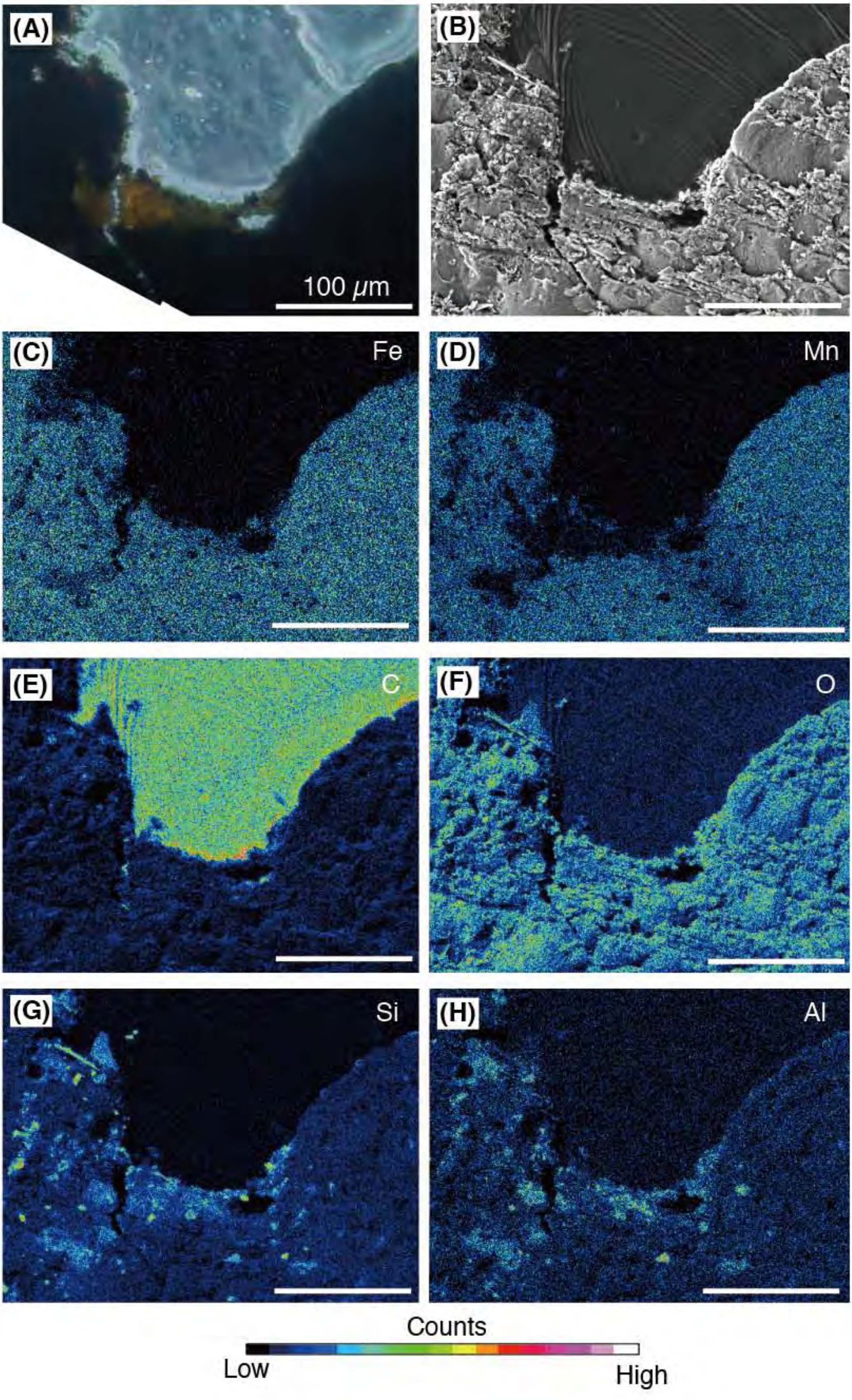

Fig. S6

679R1

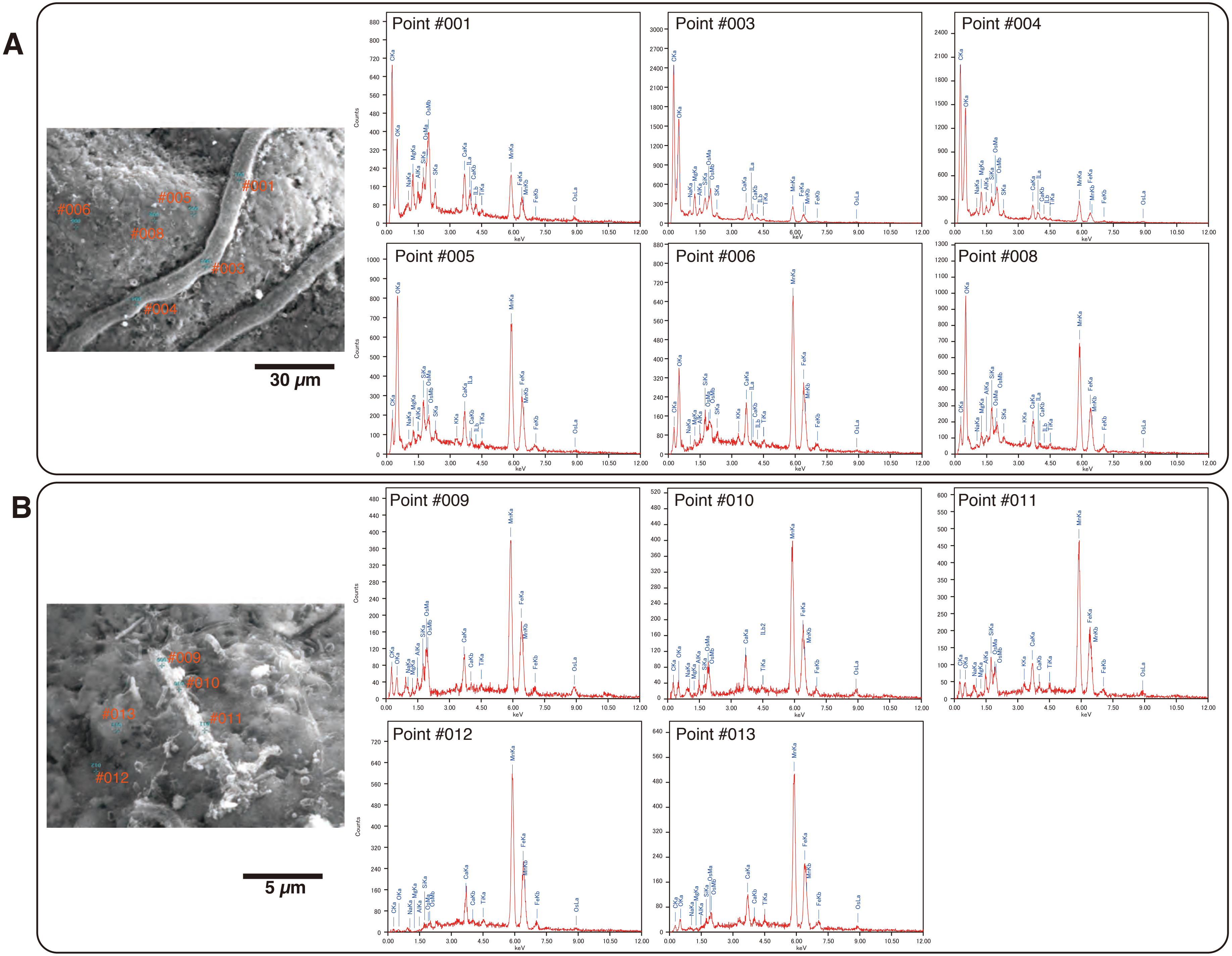

Fig. S7A, B

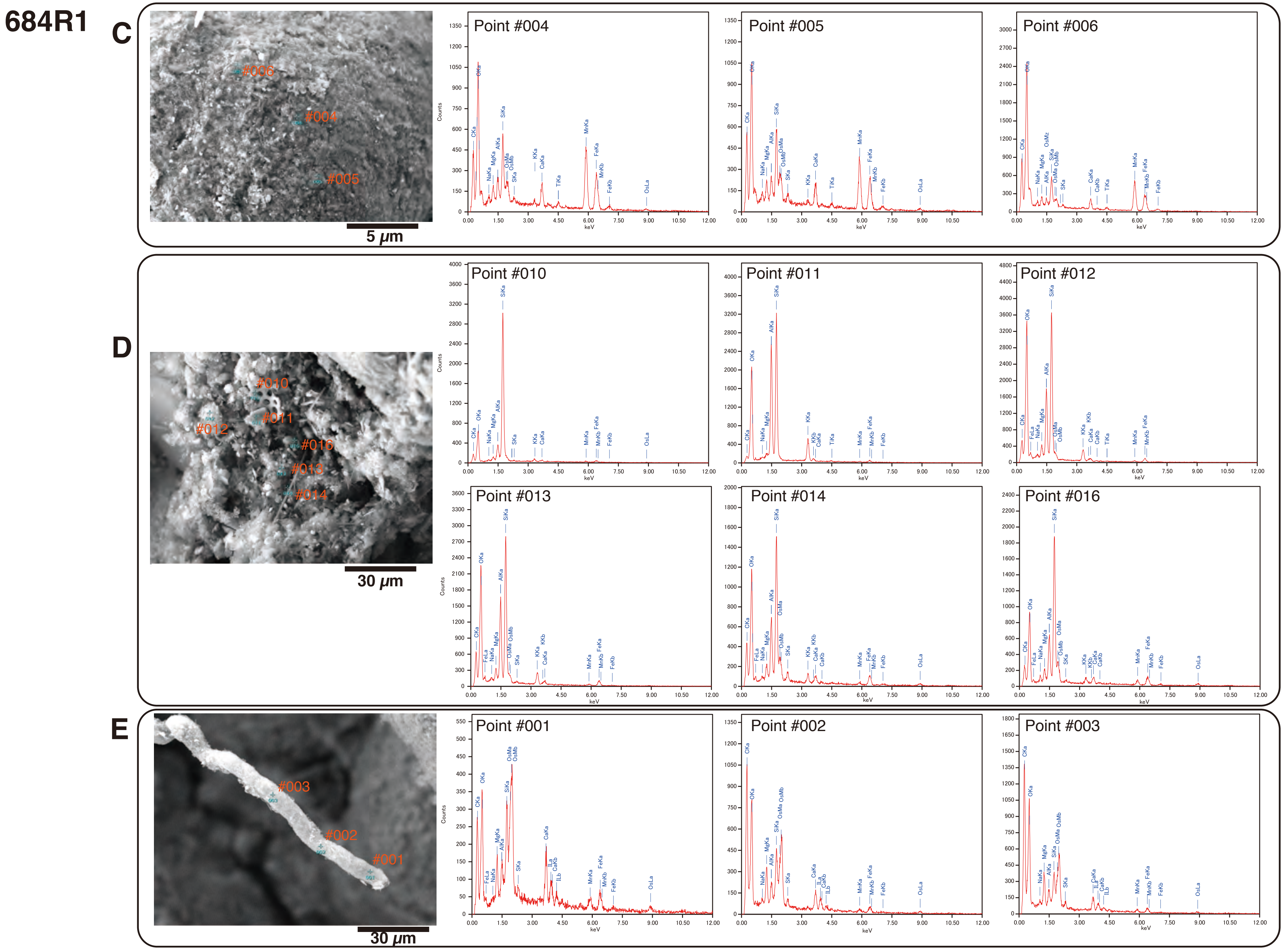
684R1
C
#004
#005
#006
5 μm
Point #004
Point #005
Point #006
D
#010
#011
#012
#013
#014
#016
30 μm
Point #010
Point #011
Point #012
Point #013
Point #014
Point #016
E
#001
#002
#003
30 μm
Point #001
Point #002
Point #003
Counts
keV

Fig. S7C-E

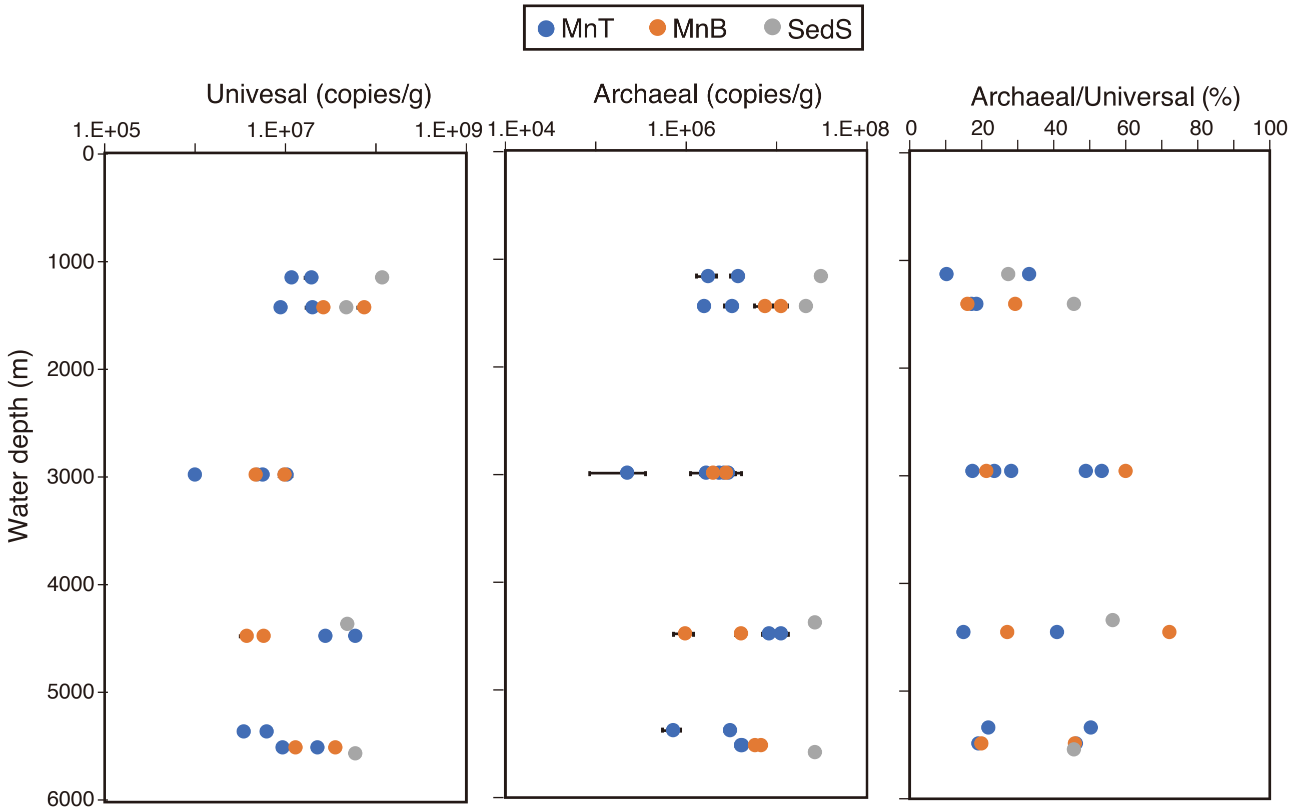

MnT
MnB
SedS
Univesal (copies/g)
1.E+05
1.E+07
1.E+09
Archaeal (copies/g)
1.E+04
1.E+06
1.E+08
Archaeal/Universal (%)
0
20
40
60
80
100
Water depth (m)
0
1000
2000
3000
4000
5000
6000

Fig. S8

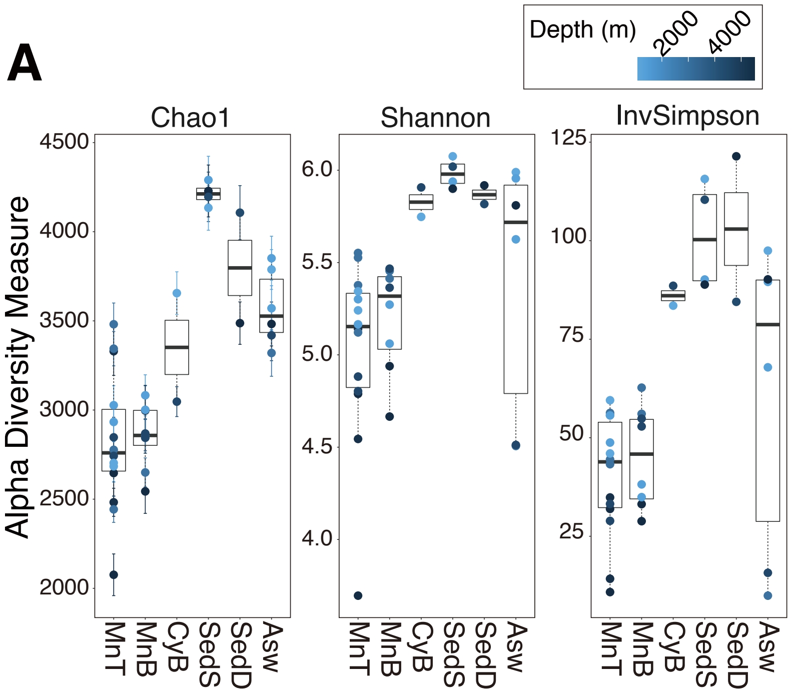

B

Chao1

	MnT	MnB	CyB	SedS	SedD
MnB	0.735	-			
CyB	0.198	0.089	-		
SedS	0.001	0.004	0.133	-	
SedD	0.022	0.044	0.667	0.133	-
Asw	0.002	0.004	0.800	0.029	0.267

Shannon

	MnT	MnB	CyB	SedS	SedD
MnB	0.792	-			
CyB	0.022	0.044	-		
SedS	0.001	0.004	0.267	-	
SedD	0.022	0.044	0.667	0.267	-
Asw	0.001	0.004	0.800	0.200	1.000

InvSimpson

	MnT	MnB	CyB	SedS	SedD
MnB	1.000	-			
CyB	0.022	0.044	-		
SedS	0.001	0.004	0.133	-	
SedD	0.022	0.044	1.000	1.000	-
Asw	0.001	0.004	0.533	0.200	0.800

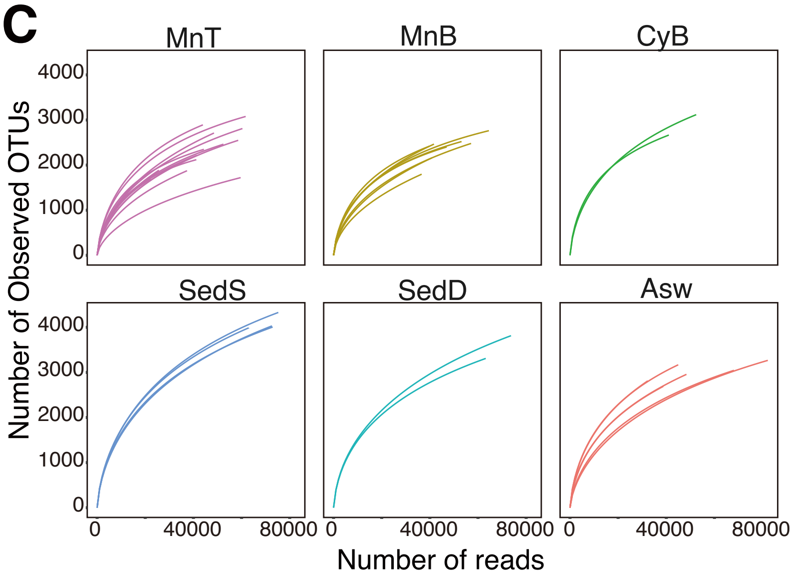

Fig. S9

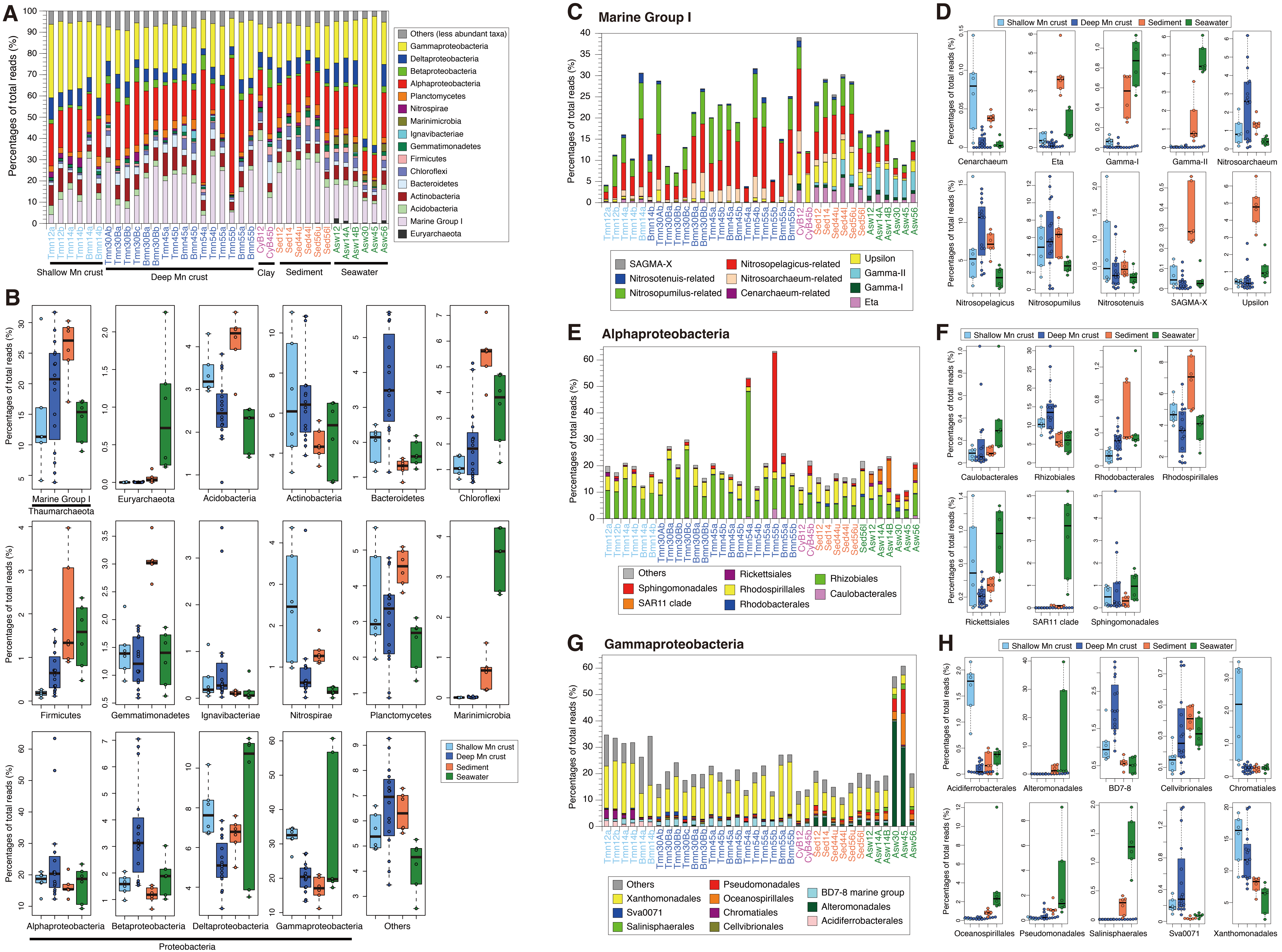
A
Percentages of total reads (%)
Others (less abundant taxa)
Gammaproteobacteria
Deltaproteobacteria
Betaproteobacteria
Alphaproteobacteria
Planctomycetes
Nitrospirae
Marinimicrobia
Ignavibacteriae
Gemmatimonadetes
Firmicutes
Chloroflexi
Bacteroidetes
Actinobacteria
Acidobacteria
Marine Group I
Euryarchaeota
Shallow Mn crust
Deep Mn crust
Clay
Sediment
Seawater
B
Marine Group I
Thaumarchaeota
Euryarchaeota
Acidobacteria
Actinobacteria
Bacteroidetes
Chloroflexi
Firmicutes
Gemmatimonadetes
Ignavibacteriae
Nitrospirae
Planctomycetes
Marinimicrobia
Alphaproteobacteria
Betaproteobacteria
Deltaproteobacteria
Gammaproteobacteria
Others
Proteobacteria
Shallow Mn crust
Deep Mn crust
Sediment
Seawater
C
Marine Group I
SAGMA-X
Nitrosotenuis-related
Nitrosopumilus-related
Nitrosopelagicus-related
Nitrosoarchaeum-related
Cenarchaeum-related
Upsilon
Gamma-II
Gamma-I
Eta
D
Cenarchaeum
Eta
Gamma-I
Gamma-II
Nitrosoarchaeum
Nitrosopelagicus
Nitrosopumilus
Nitrosotenuis
SAGMA-X
Upsilon
E
Alphaproteobacteria
Others
Sphingomonadales
SAR11 clade
Rickettsiales
Rhodospirillales
Rhodobacterales
Rhizobiales
Caulobacterales
F
Caulobacterales
Rhizobiales
Rhodobacterales
Rhodospirillales
Rickettsiales
SAR11 clade
Sphingomonadales
G
Gammaproteobacteria
Others
Xanthomonadales
Sva0071
Salinisphaerales
Pseudomonadales
Oceanospirillales
Chromatiales
Cellvibrionales
BD7-8 marine group
Alteromonadales
Acidiferrobacterales
H
Acidiferrobacterales
Alteromonadales
BD7-8
Cellvibrionales
Chromatiales
Oceanospirillales
Pseudomonadales
Salinisphaerales
Sva0071
Xanthomonadales

Fig. S10

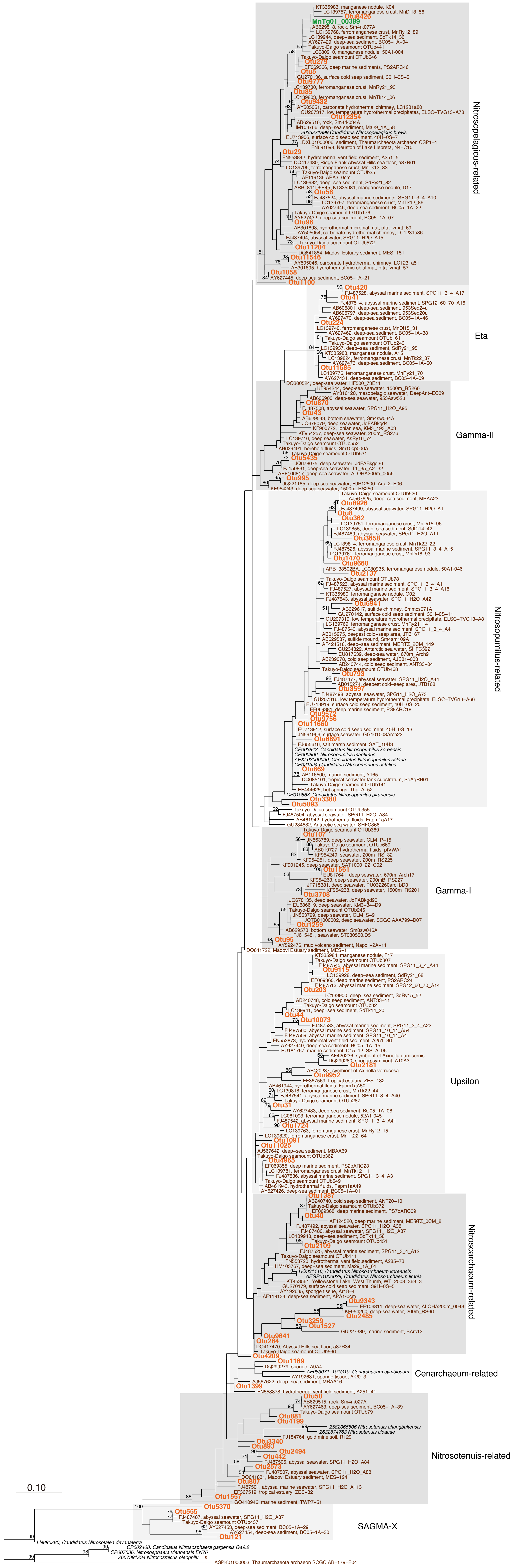
Nitrosopelagicus-related
Eta
Gamma-II
Nitrosopumilus-related
Gamma-I
Upsilon
Nitrosoarchaeum-related
Cenarchaeum-related
Nitrosotenuis-related
SAGMA-X
0.10

Fig. S11

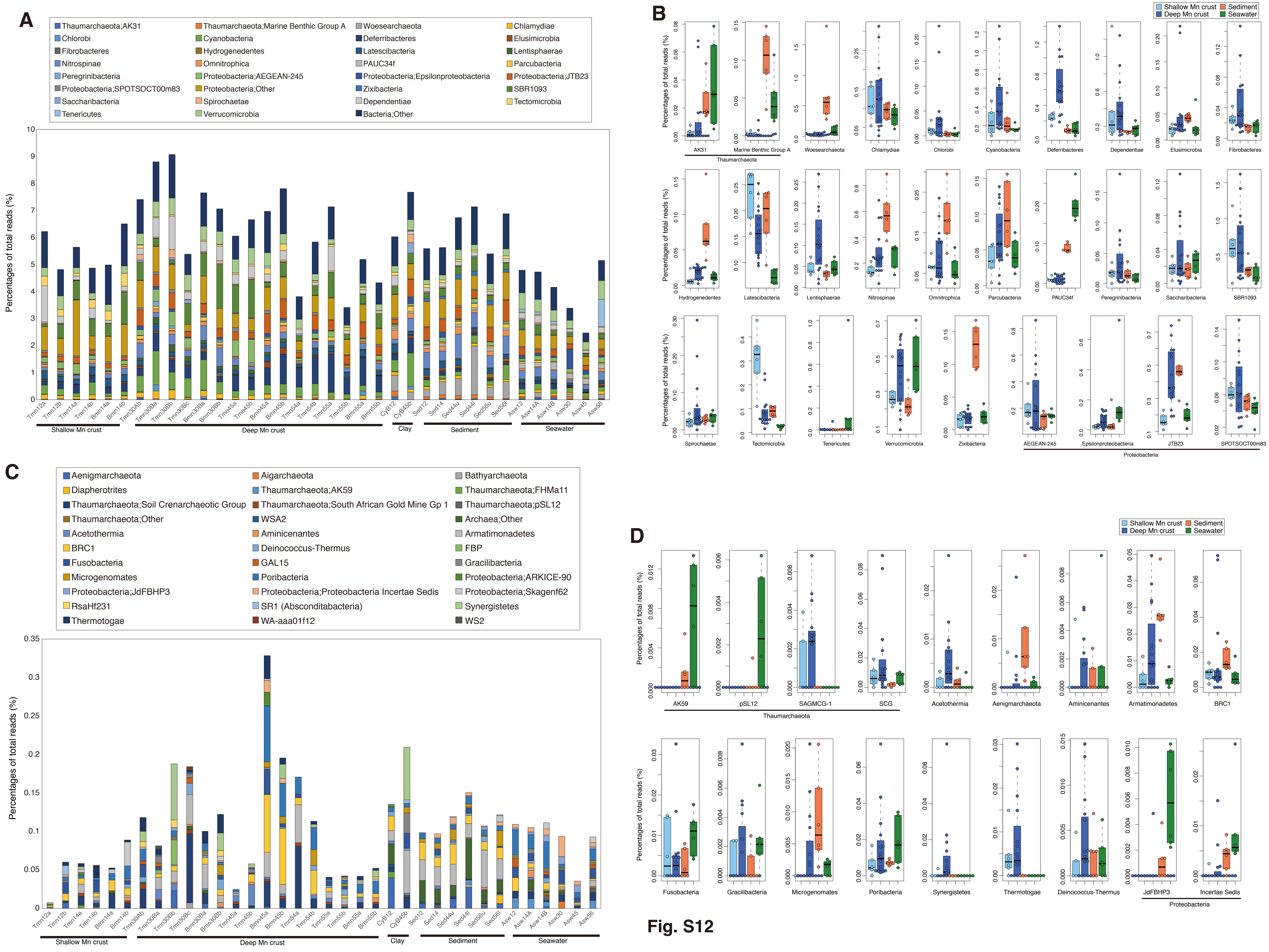
A
Thaumarchaeota;AK31
Chlorobi
Fibrobacteres
Nitrospinae
Peregrinibacteria
Proteobacteria;SPOTSOCT00m83
Saccharibacteria
Tenericutes
Thaumarchaeota;Marine Benthic Group A
Cyanobacteria
Hydrogenedentes
Omnitrophica
Proteobacteria;AEGEAN-245
Proteobacteria;Other
Spirochaetae
Verrucomicrobia
Woesearchaeota
Deferribacteres
Latescibacteria
PAUC34f
Proteobacteria;Epsilonproteobacteria
Zixibacteria
Dependentiae
Bacteria;Other
Chlamydiae
Elusimicrobia
Lentisphaerae
Parcubacteria
Proteobacteria;JTB23
SBR1093
Tectomicrobia
Percentages of total reads (%)
Shallow Mn crust
Deep Mn crust
Clay
Sediment
Seawater
B
Shallow Mn crust
Deep Mn crust
Sediment
Seawater
Percentages of total reads (%)
AK31
Marine Benthic Group A
Thaumarchaeota
Woesearchaeota
Chlamydiae
Chlorobi
Cyanobacteria
Deferribacteres
Dependentiae
Elusimicrobia
Fibrobacteres
Hydrogenedentes
Latescibacteria
Lentisphaerae
Nitrospinae
Omnitrophica
Parcubacteria
PAUC34f
Peregrinibacteria
Saccharibacteria
SBR1093
Spirochaetae
Tectomicrobia
Tenericutes
Verrucomicrobia
Zixibacteria
AEGEAN-245
Epsilonproteobacteria
JTB23
SPOTSOCT00m83
Proteobacteria
C
Aenigmarchaeota
Diapherotrites
Thaumarchaeota;Soil Crenarchaeotic Group
Thaumarchaeota;Other
Acetothermia
BRC1
Fusobacteria
Microgenomates
Proteobacteria;JdFBHP3
RsaHf231
Thermotogae
Aigarchaeota
Thaumarchaeota;AK59
Thaumarchaeota;South African Gold Mine Gp 1
WSA2
Aminicenantes
Deinococcus-Thermus
GAL15
Poribacteria
Proteobacteria;Proteobacteria Incertae Sedis
SR1 (Absconditabacteria)
WA-aaa01f12
Bathyarchaeota
Thaumarchaeota;FHMa11
Thaumarchaeota;pSL12
Archaea;Other
Armatimonadetes
FBP
Gracilibacteria
Proteobacteria;ARKICE-90
Proteobacteria;Skagenf62
Synergistetes
WS2
Percentages of total reads (%)
Shallow Mn crust
Deep Mn crust
Clay
Sediment
Seawater
D
Shallow Mn crust
Deep Mn crust
Sediment
Seawater
Percentages of total reads (%)
AK59
pSL12
SAGMCG-1
SCG
Thaumarchaeota
Acetothermia
Aenigmarchaeota
Aminicenantes
Armatimonadetes
BRC1
Fusobacteria
Gracilibacteria
Microgenomates
Poribacteria
Synergistetes
Thermotogae
Deinococcus-Thermus
JdFBHP3
Incertae Sedis
Proteobacteria

Fig. S12

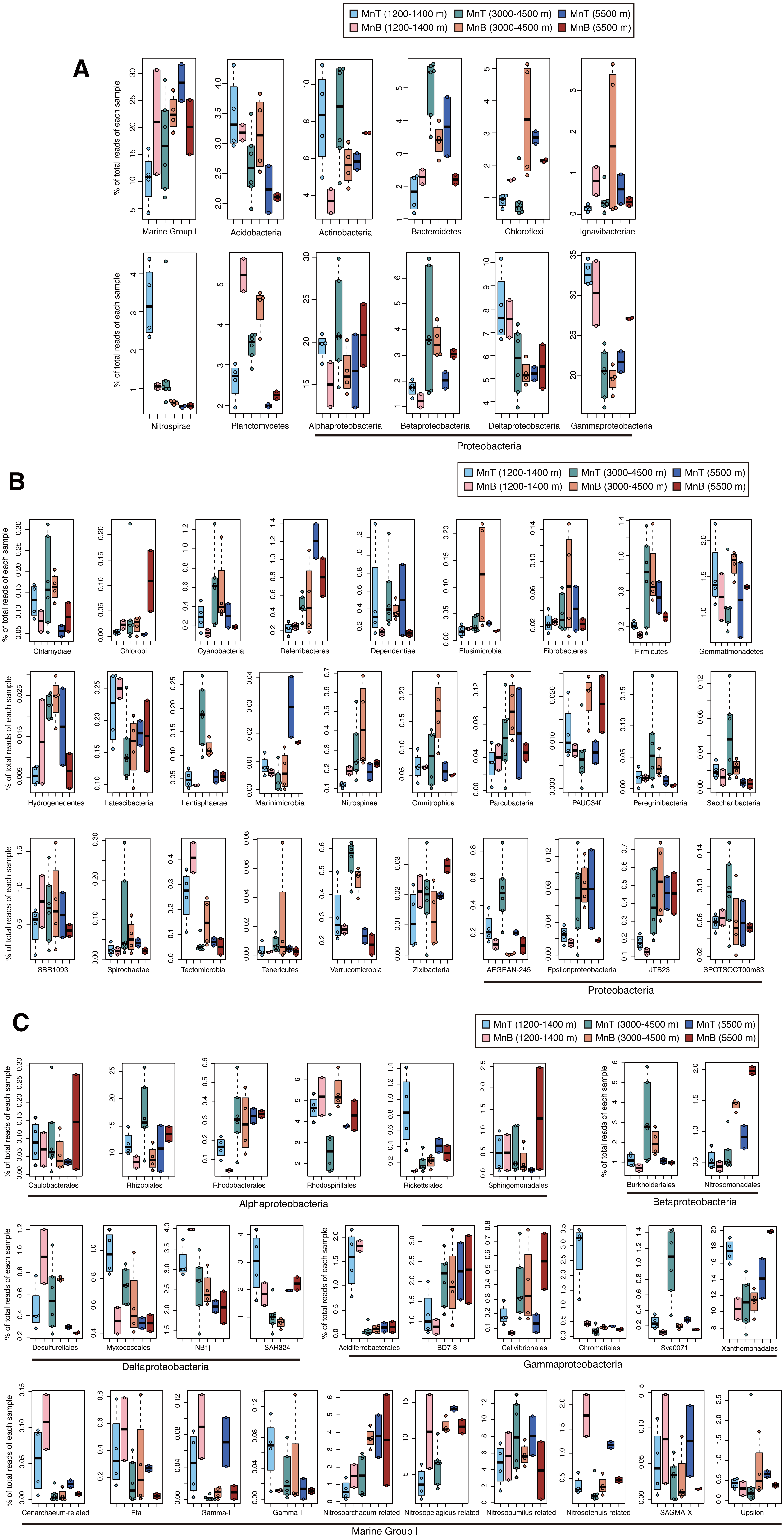
MnT (1200-1400 m)
MnT (3000-4500 m)
MnT (5500 m)
MnB (1200-1400 m)
MnB (3000-4500 m)
MnB (5500 m)
A
% of total reads of each sample
Marine Group I
Acidobacteria
Actinobacteria
Bacteroidetes
Chloroflexi
Ignavibacteriae
Nitrospirae
Planctomycetes
Alphaproteobacteria
Betaproteobacteria
Deltaproteobacteria
Gammaproteobacteria
Proteobacteria
B
Chlamydiae
Chlorobi
Cyanobacteria
Deferribacteres
Dependentiae
Elusimicrobia
Fibrobacteres
Firmicutes
Gemmatimonadetes
Hydrogenedentes
Latescibacteria
Lentisphaerae
Marinimicrobia
Nitrospinae
Omnitrophica
Parcubacteria
PAUC34f
Peregrinibacteria
Saccharibacteria
SBR1093
Spirochaetae
Tectomicrobia
Tenericutes
Verrucomicrobia
Zixibacteria
AEGEAN-245
Epsilonproteobacteria
JTB23
SPOTSOCT00m83
Proteobacteria
C
Caulobacterales
Rhizobiales
Rhodobacterales
Rhodospirillales
Rickettsiales
Sphingomonadales
Alphaproteobacteria
Burkholderiales
Nitrosomonadales
Betaproteobacteria
Desulfurellales
Myxococcales
NB1j
SAR324
Deltaproteobacteria
Acidiferrobacterales
BD7-8
Cellvibrionales
Chromatiales
Sva0071
Xanthomonadales
Gammaproteobacteria
Cenarchaeum-related
Eta
Gamma-I
Gamma-II
Nitrosoarchaeum-related
Nitrosopelagicus-related
Nitrosopumilus-related
Nitrosotenuis-related
SAGMA-X
Upsilon
Marine Group I

Fig. S13

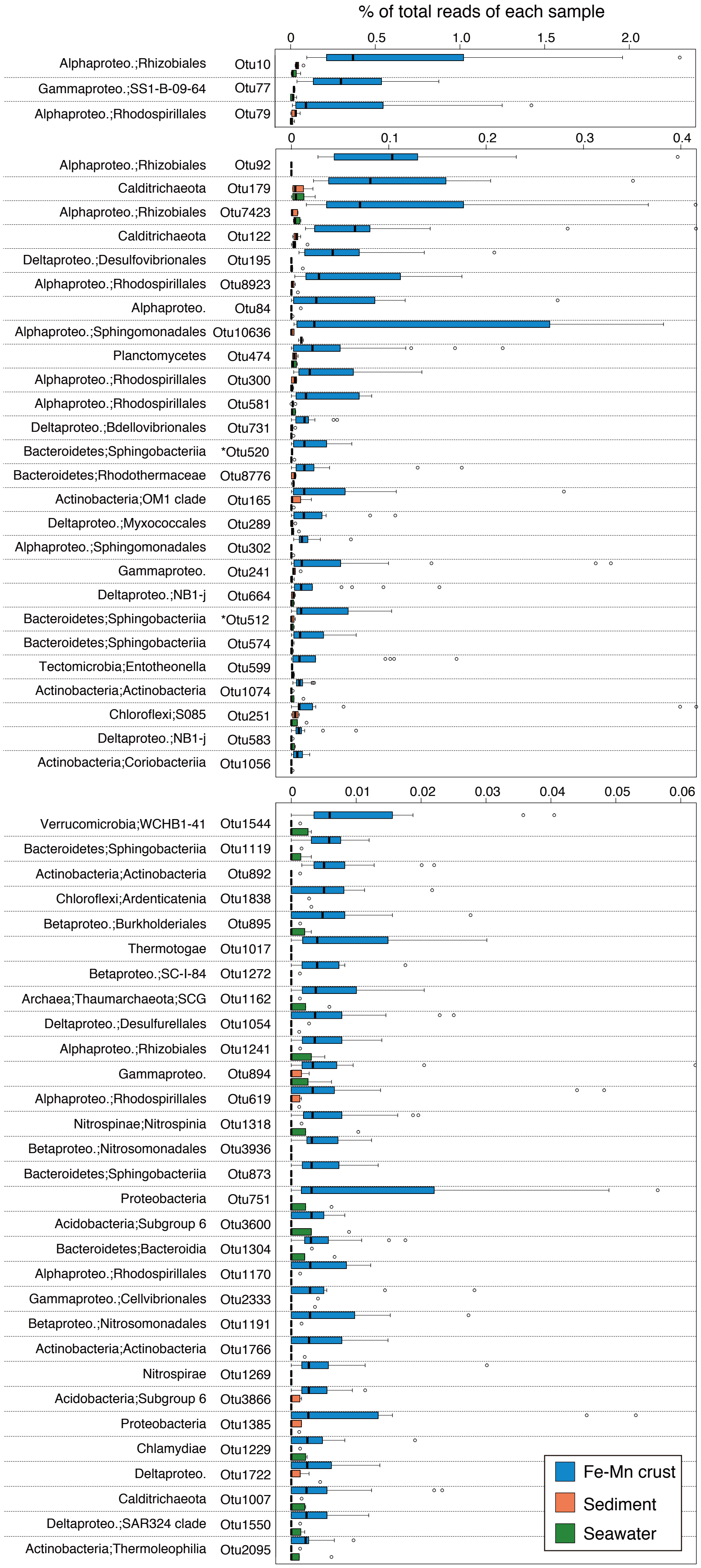

% of total reads of each sample
0
0.5
1.0
1.5
2.0
Alphaproteo.;Rhizobiales Otu10
Gammaproteo.;SS1-B-09-64 Otu77
Alphaproteo.;Rhodospirillales Otu79
0
0.1
0.2
0.3
0.4
Alphaproteo.;Rhizobiales Otu92
Calditrichaeota Otu179
Alphaproteo.;Rhizobiales Otu7423
Calditrichaeota Otu122
Deltaproteo.;Desulfovibrionales Otu195
Alphaproteo.;Rhodospirillales Otu8923
Alphaproteo. Otu84
Alphaproteo.;Sphingomonadales Otu10636
Planctomycetes Otu474
Alphaproteo.;Rhodospirillales Otu300
Alphaproteo.;Rhodospirillales Otu581
Deltaproteo.;Bdellovibrionales Otu731
Bacteroidetes;Sphingobacteriia *Otu520
Bacteroidetes;Rhodothermaceae Otu8776
Actinobacteria;OM1 clade Otu165
Deltaproteo.;Myxococcales Otu289
Alphaproteo.;Sphingomonadales Otu302
Gammaproteo. Otu241
Deltaproteo.;NB1-j Otu664
Bacteroidetes;Sphingobacteriia *Otu512
Bacteroidetes;Sphingobacteriia Otu574
Tectomicrobia;Entotheonella Otu599
Actinobacteria;Actinobacteria Otu1074
Chloroflexi;S085 Otu251
Deltaproteo.;NB1-j Otu583
Actinobacteria;Coriobacteriia Otu1056
0
0.01
0.02
0.03
0.04
0.05
0.06
Verrucomicrobia;WCHB1-41 Otu1544
Bacteroidetes;Sphingobacteriia Otu1119
Actinobacteria;Actinobacteria Otu892
Chloroflexi;Ardenticatenia Otu1838
Betaproteo.;Burkholderiales Otu895
Thermotogae Otu1017
Betaproteo.;SC-I-84 Otu1272
Archaea;Thaumarchaeota;SCG Otu1162
Deltaproteo.;Desulfurellales Otu1054
Alphaproteo.;Rhizobiales Otu1241
Gammaproteo. Otu894
Alphaproteo.;Rhodospirillales Otu619
Nitrospinae;Nitrospinia Otu1318
Betaproteo.;Nitrosomonadales Otu3936
Bacteroidetes;Sphingobacteriia Otu873
Proteobacteria Otu751
Acidobacteria;Subgroup 6 Otu3600
Bacteroidetes;Bacteroidia Otu1304
Alphaproteo.;Rhodospirillales Otu1170
Gammaproteo.;Cellvibrionales Otu2333
Betaproteo.;Nitrosomonadales Otu1191
Actinobacteria;Actinobacteria Otu1766
Nitrospirae Otu1269
Acidobacteria;Subgroup 6 Otu3866
Proteobacteria Otu1385
Chlamydiae Otu1229
Deltaproteo. Otu1722
Calditrichaeota Otu1007
Deltaproteo.;SAR324 clade Otu1550
Actinobacteria;Thermoleophilia Otu2095
Fe-Mn crust
Sediment
Seawater

Fig. S14

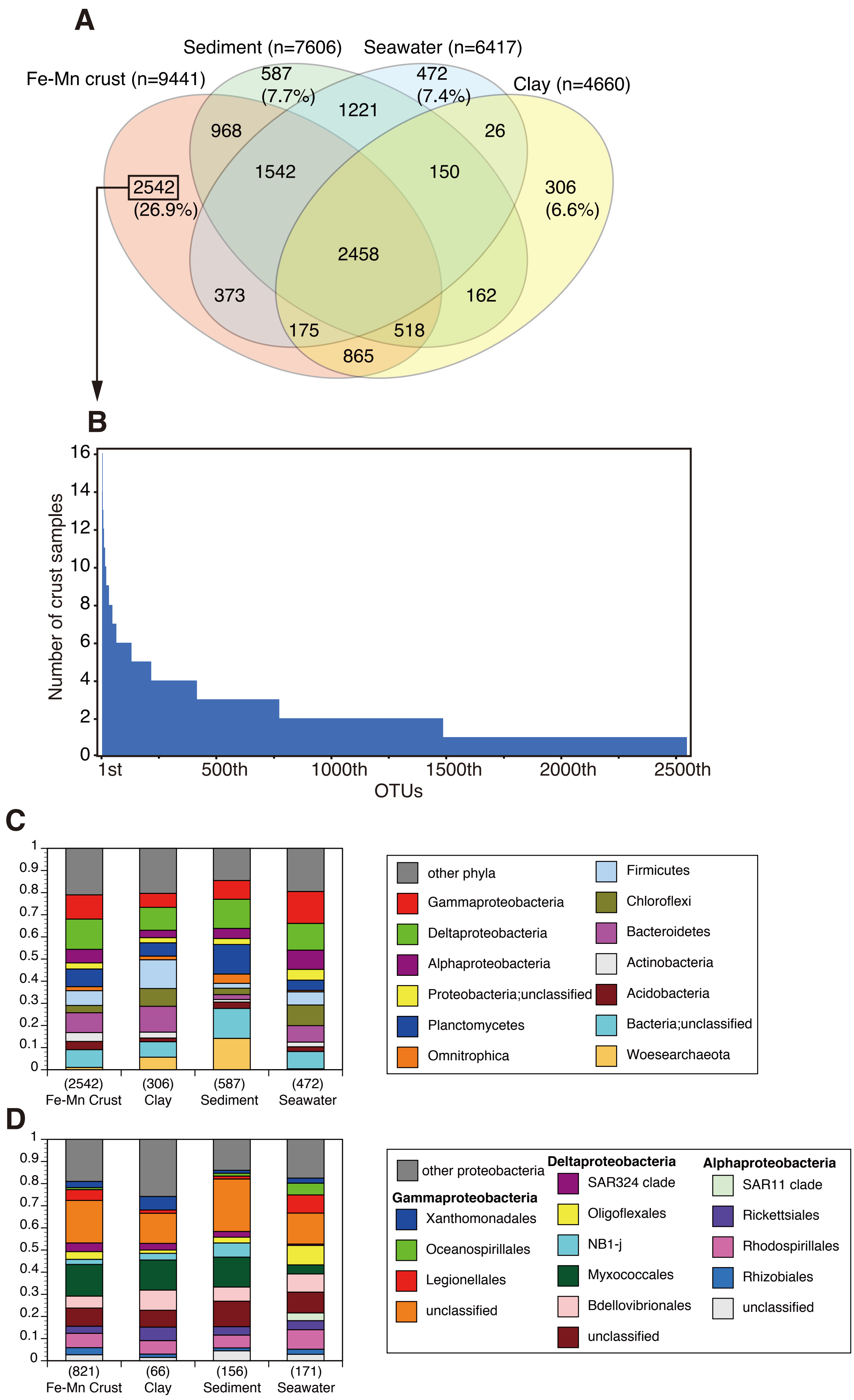
A
Fe-Mn crust (n=9441)
Sediment (n=7606)
Seawater (n=6417)
Clay (n=4660)
2542
(26.9%)
587
(7.7%)
472
(7.4%)
306
(6.6%)
968
1221
26
1542
150
2458
373
162
175
518
865
B
Number of crust samples
OTUs
1st
500th
1000th
1500th
2000th
2500th
C
other phyla
Gammaproteobacteria
Deltaproteobacteria
Alphaproteobacteria
Proteobacteria;unclassified
Planctomycetes
Omnitrophica
Firmicutes
Chloroflexi
Bacteroidetes
Actinobacteria
Acidobacteria
Bacteria;unclassified
Woesearchaeota
(2542) Fe-Mn Crust
(306) Clay
(587) Sediment
(472) Seawater
D
other proteobacteria
Gammaproteobacteria
Xanthomonadales
Oceanospirillales
Legionellales
unclassified
Deltaproteobacteria
SAR324 clade
Oligoflexales
NB1-j
Myxococcales
Bdellovibrionales
unclassified
Alphaproteobacteria
SAR11 clade
Rickettsiales
Rhodospirillales
Rhizobiales
unclassified
(821) Fe-Mn Crust
(66) Clay
(156) Sediment
(171) Seawater

Fig. S15

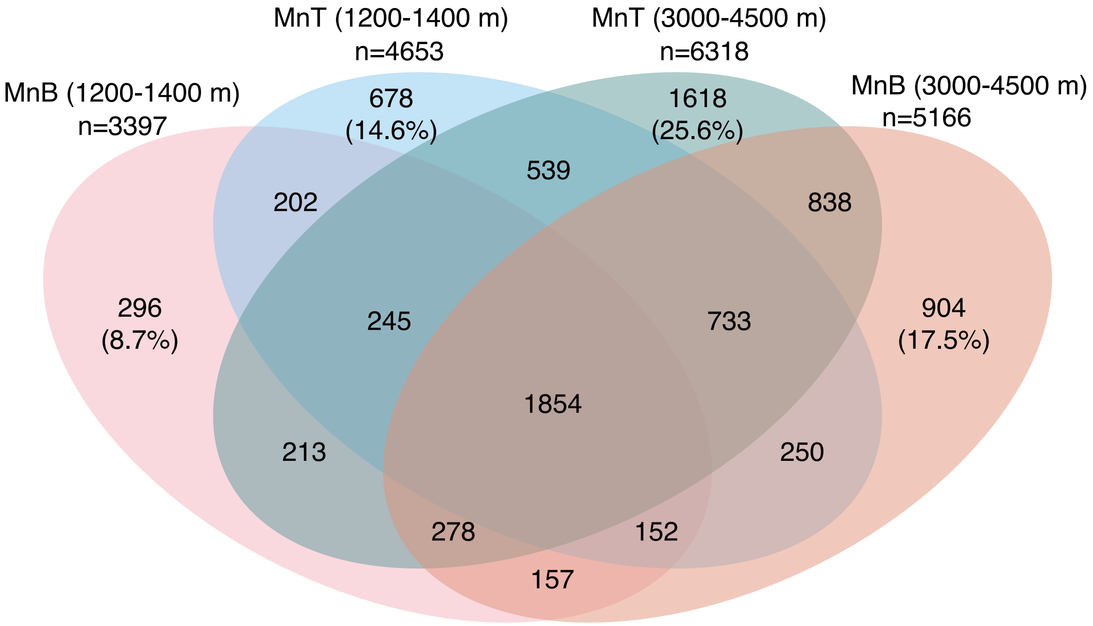
MnT (1200-1400 m)
n=4653
MnT (3000-4500 m)
n=6318
MnB (1200-1400 m)
n=3397
MnB (3000-4500 m)
n=5166
678
(14.6%)
1618
(25.6%)
539
202
838
296
(8.7%)
245
733
904
(17.5%)
1854
213
250
278
152
157

Fig. S16
